# DNA hypermethylation-induced miR-182 silence targets BCL2 and HOXA9 to facilitate the self-renewal of leukemia stem cell, accelerate acute myeloid leukemia progression, and determine the sensitivity of BCL2 inhibitor venetoclax

**DOI:** 10.7150/thno.77404

**Published:** 2023-01-01

**Authors:** Sisi Ye, Fang Xiong, Xiaofei He, Yigang Yuan, Danyang Li, Daijiao Ye, Liuzhi Shi, Zihan Lin, Min Zhao, Shuya Feng, Bin Zhou, Huachun Weng, Lili Hong, Haige Ye, Shenmeng Gao

**Affiliations:** 1 Medical Research Center, The First Affiliated Hospital of Wenzhou Medical University, 1 Xuefubei Street, Ouhai District, Wenzhou, Zhejiang Province, China.; 2 Department of Clinical Laboratory, The First Affiliated Hospital of Wenzhou Medical University, 1 Xuefubei Street, Ouhai District, Wenzhou, Zhejiang Province, China.; 3 The College of Medical Technology, Shanghai University of Medicine& Health Sciences; 279 ZhouZhuGong Street, Pudong District, Shanghai, China.; 4 Department of Hematology, The First Affiliated Hospital of Zhejiang Chinese Medical University, 54 Post Road, Hangzhou, Zhejiang Province, China.; 5 Department of Hematology, The First Affiliated Hospital of Wenzhou Medical University, 1 Xuefubei Street, Ouhai District, Wenzhou, Zhejiang Province, China.

**Keywords:** Hypermethylation, MicroRNA, Leukemia stem cell, Venetoclax

## Abstract

**Rationale:** microRNAs (miRNAs) are frequently deregulated and play important roles in the pathogenesis and progression of acute myeloid leukemia (AML). miR-182 functions as an onco-miRNA or tumor suppressor miRNA in the context of different cancers. However, whether miR-182 affects the self-renewal of leukemia stem cells (LSCs) and normal hematopoietic stem progenitor cells (HSPCs) is unknown.

**Methods:** Bisulfite sequencing was used to analyze the methylation status at pri-miR-182 promoter. Lineage-negative HSPCs were isolated from miR-182 knockout (182KO) and wild-type (182WT) mice to construct MLL-AF9-transformed AML model. The effects of miR-182 depletion on the overall survival and function of LSC were analyzed in this mouse model *in vivo*.

**Results:** miR-182-5p (miR-182) expression was lower in AML blasts than normal controls (NCs) with hypermethylation observed at putative pri-miR-182 promoter in AML blasts but unmethylation in NCs. Overexpression of miR-182 inhibited proliferation, reduced colony formation, and induced apoptosis in leukemic cells. In addition, depletion of miR-182 accelerated the development and shortened the overall survival (OS) in MLL-AF9-transformed murine AML through increasing LSC frequency and self-renewal ability. Consistently, overexpression of miR-182 attenuated AML development and extended the OS in the murine AML model. Most importantly, miR-182 was likely dispensable for normal hematopoiesis. Mechanistically, we identified BCL2 and HOXA9 as two key targets of miR-182 in this context. Most importantly, AML patients with miR-182 unmethylation had high expression of miR-182 followed by low protein expression of BCL2 and resistance to BCL2 inhibitor venetoclax (Ven) *in vitro*.

**Conclusions:** Our results suggest that miR-182 is a potential therapeutic target for AML patients through attenuating the self-renewal of LSC but not HSPC. miR-182 promoter methylation could determine the sensitivity of Ven treatment and provide a potential biomarker for it.

## Introduction

Acute myeloid leukemia (AML), the most common hematologic malignancy in adults, derives from hematopoietic stem and progenitor cells (HSPCs) because of the accumulation of various genetic and epigenetic alterations 1. Although the standard chemotherapy, bone marrow (BM) transplantation (BMT), and immunotherapy have substantially improved the overall survival (OS) time, less than 50% of AML patients survive more than five years 2. Accumulating evidences have indicated that activation of oncogenic proteins results in the uncontrolled growth of immature myeloid blasts and block of differentiation 3. For example, mixed-lineage leukemia (MLL) fusions (t(11q23)) and AML1-ETO (t(8;21)), as well as oncogenic proteins HOXA9 and BCL2, facilitate the self-renewal ability and inhibit the apoptosis of AML blasts 4, 5. Leukemia stem cell (LSC) is considered to be responsible for the initiation, maintenance, chemotherapy resistance, and relapse of AML. In addition, LSC is hard to be completely eradicated by therapeutic strategies, including chemotherapy, BMT, and radiotherapy 6. However, LSC has some similarities with normal HSPC. Therefore, new therapeutic methods that target leukemia-initiating cell (LIC)/LSC with low toxicity against HSPC are urgently needed for AML patients.

miRNA is a conserved 19-21 nucleotides non-coding RNA, which regulates the expression of target genes by directly binding to the 3'-untranslated region (UTR) of mRNAs, leading to the degradation of mRNA or translational suppression 7. Several studies have demonstrated that miRNA functions as either a tumor suppressor or an onco-miRNA in various subtypes of AML through regulating leukemic processes, such as survival, self-renewal, chemotherapy resistance, and leukemia initiation 8. For example, miR-126 and miR-17-92 cluster maintains the self-renewal of LSC and facilitates the development of MLL-associated leukemia 9, 10. In addition, miRNA supports and regulates normal HSPC function 11, 12. However, most miRNAs both affect the function of HSPC and LSC 13, 14, suggesting that these miRNAs are not suitable for anti-LSC therapy. Therefore, miRNAs that can modulate the self-renewal ability of LSC but not HSPC have potential application for AML patients.

The deregulation of miRNA in AML blasts is mainly caused by the deletion of the miRNA-coding gene, copy number amplifications, and epigenetic alterations 15. Hypermethylation of CpG islands within promoter region, which leads to the tumor-specific decrease of miRNA, has been reported in the initiation and development of AML 16. Hypermethylation in the proximal promoter regions of miR-193a 16, miR-143 17, and miR-34a 18 results in the decreased expression of miRNAs in AML blasts and facilitates the initiation of AML. Hypomethylation agent (HMA) 5-Azacytidine (AZA) increases the expression of miRNAs, followed by the reduction of miRNA-target genes 17, 19. However, whether miRNA promoter methylation is associated with the response of HMA is largely unknown.

MicroRNA-182-5p (miR-182), located at chromosome 7q32.2, has been reported as an onco-miRNA in cervical cancer, prostate cancer, and breast cancer, as well as tumor suppressor miRNA in colon cancer, lung cancer, and gastric adenocarcinoma 20-25. *VEGF-C*, *cortactin*, *CREB1* and* Rad51* are potential targets of miR-182 25-28. Although several studies have also reported that miR-182 mediates chemotherapy sensitivity and leukemic differentiation 28, 29, the role of miR-182 in the self-renewal ability of LSC and normal hematopoiesis remains elusive. In this study, we comprehensively identified the function of miR-182 as a tumor suppressor in AML and explored miR-182 promoter methylation as a predictive marker for Ven treatment.

## Material and methods

### Cell lines, primary AML blasts, and normal CD34^+^ HSPCs

Human leukemic cell lines, including MOLM-13 and THP1 (ATCC, Manassas, VA, USA), were cultured in humidified 37 °C incubator with 5% CO_2_ in RPMI 1640 with 10% fetal bovine serum (FBS, Invitrogen, Carlsbad, CA, USA) and 1% penicillin streptomycin. BM blasts were isolated by Ficoll density gradient centrifugation (GE Healthcare, Uppsala, Sweden) from untreated AML patients. Human normal CD34^+^ cells were isolated by the EasySep™ human CD34 positive selection kit (StemCell Technologies, Vancouver, BC, Canada) from healthy volunteers. Primary AML blasts and normal CD34^+^ HSPCs were preserved in liquid nitrogen until use. Primary AML blasts were cultured in StemSpan Serum-Free Expansion Medium (SFEM; Stemcell Technologies) supplemented with recombinant human stem cell factor (SCF, PeproTech, Rocky Hill, NJ, USA), interleukin-3 (IL-3, PeproTech), interleukin-6 (IL-6, PeproTech) at 10 ng/mL each. Normal CD34^+^ cells were cultured in SFEM (Stemcell Technologies) supplemented with human thrombopoietin (TPO, PeproTech), fms related receptor tyrosine kinase 3 ligand (Flt3 ligand, PeproTech), and SCF (PeproTech) at 100 ng/mL each. According to the declaration of Helsinki, both AML patients and healthy volunteers have provided informed consent. All procedures in our studies involving AML patients and healthy volunteers followed the Declaration of Helsinki and the Ethics Committee of the First Affiliated Hospital of Wenzhou Medical University. The clinical characteristics of AML patients are summarized in Table S1.

### RNA extraction and quantitative RT-PCR (qRT-PCR)

Total mRNA and miRNA from human leukemic cells and murine BM cells were extracted by Trizol reagent with minor modifications 30. After extraction, RNA concentration and quality were determined by measuring the absorbance at 260/280 nm (DS-11 spectrophotometer, DeNovix, Wilmington, DE, USA). cDNA for qRT-PCR analysis was synthesized by using a Q5 real-time PCR system (Applied Biosystems, Carlsbad, CA, USA). miR-182 and U6 small nuclear RNA (snRNA) were reversely transcribed using Stem-loop RT primer with PrimeScript™ RT Master Mix (Takara Bio, Tokyo, Japan). U6 snRNA was used as an endogenous control for qPCR with miR-182. The special primers for miR-182 and U6 were provided by RIBOBIO company (Guangzhou, China). Human GAPDH and murine β-actin were used as endogenous controls for qRT-PCR of human and murine samples, respectively. SYBR Green dye (Takara) was used to determine the expression of mRNA and miRNA. Relative expression was calculated using the 2^-ΔΔCT^ method. All of the primer sequences were shown in Table S2.

### Analysis of target genes

The following three major miRNA-target prediction programs were used to predict putative miRNA-target pairs: TargetScan (http://www.targetscan.org) 31, miRwalk 32, and miRanda 33.

### MLL-AF9 and AML1-ETO(9a)-induced murine AML model

BM lineage-negative (Lin^-^) cells were isolated from 8-week-old 182WT and 182KO mice (Stemcell Technologies) five days after 5-fluorouracil treatment and cultured in StemSpan SFEM (Stemcell Technologies) supplemented with murine SCF (50 ng/mL, PeproTech), TPO (50 ng/mL, PeproTech), and FLT3 ligand (50 ng/mL, PeproTech) overnight. Furthermore, Lin^-^ cells were retrovirally transduced with MSCV-green fluorescent protein (GFP)-internal ribosome entry site (IRES)-MLL-AF9 4 or MSCV-GFP-IRES-AML1-ETO(9a) 34 by two rounds of spinoculation at 2000×rpm for 2 h 35. GFP^+^ cells were sorted by fluorescence-activated cell sorting and were intravenously injected into lethally irradiated C57BL/6J mice (Beijing Vital River Laboratory, Beijing, China) plus radioprotective BM cells (4×10^5^ for every mouse). The sample size *in vivo* study was estimated according to prior experience in our laboratory. All animal procedures and care are performed according to national and international policies and institutional guidelines of the ethics committee of the First Affiliated Hospital of Wenzhou Medical University.

### Limiting dilution assays

BM Green fluorescent protein^+^ (GFP^+^) cells were sorted by flow cytometry from secondary MLL-AF9-transduced AML mice. Three doses of donor cells from 182WT or 182KO-AML blasts were transplanted into lethally irradiated recipients (n = 6 for each group). The numbers of recipient mice were counted only when the mice developed full-blown leukemia and died within 6 months after transplantation (Table S3). Extreme limiting dilution assay (ELDA) software was used to determine LSC frequency 36.

### Other procedures

Western blot, Colony formation assay, Cell proliferation and apoptosis assays, Luciferase reporter, Competitive reconstitution assays, RNA sequencing (RNA-seq), Cell cycle, Construction of plasmids, Lentivirus and retrovirus production and transduction, Construction of plasmids, Hematological and histological analysis, Flow cytometry analysis and DNA methylation detection please see supplemental material and methods.

### Statistical analysis

All the results were expressed as Mean ± SD where applicable. The significance of the difference between groups was determined by Student's t-test. Survival was estimated according to the method of Kaplan and Meier. The log rank test was used to assess statistical significance. A *P* value of less than 0.05 was considered statistically significant. All statistical analyses were performed with SPSS software (SPSS 22.0, Chicago, IL, USA) and Prism version 5.0.

## Results

### miR-182 is low expressed in AML and associated with poor outcomes

**Figure 1:**
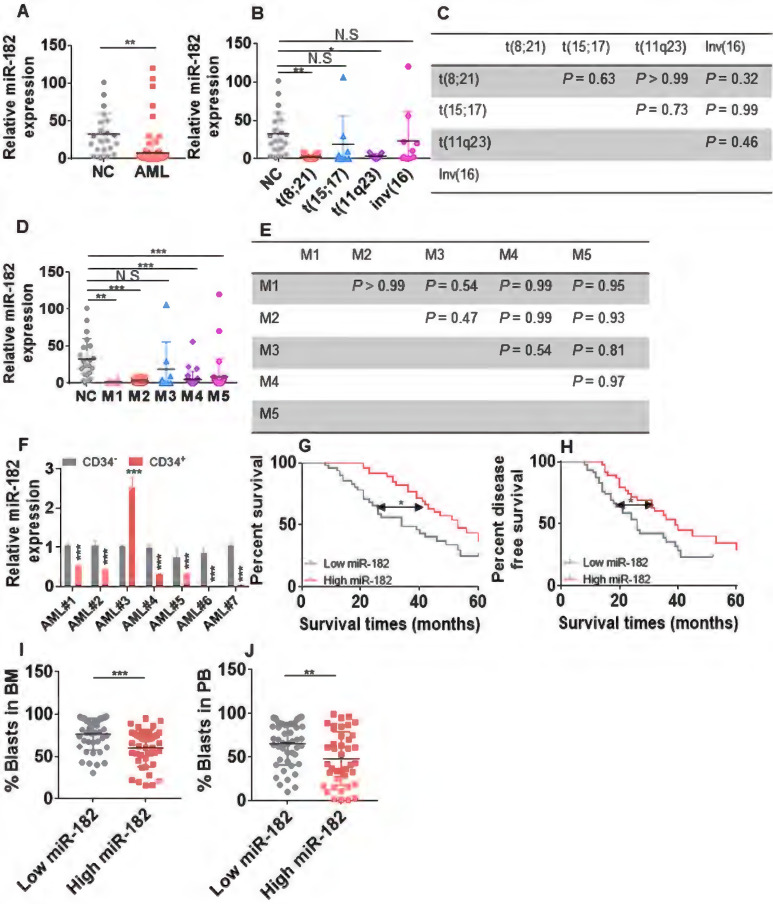
** Expression of miR-182 is lower in primary AML blasts than normal controls (NCs).** (**A**) The expressions of miR-182 were measured in bone marrow (BM) mononuclear cells from untreated AML patients and NCs by qRT-PCR. (**B and C**) The expressions of miR-182 were analyzed in untreated AML patients bearing various chromosomal translocations in comparison to NCs. (**D and E**) The expressions of miR-182 were analyzed in untreated AML patients according to FAB subtypes (M1-M5) compared with NCs. (**F**) The expressions of miR-182 were measured in CD34^+^ and CD34^-^ BM cells isolated from seven AML patients. (**G and H**) Overall survival time (OS, G) and disease free-survival time (DFS, H) were analyzed in AML patients with higher (above the median miR-182 expression) levels of miR-182 in comparison to those with lower (below the median miR-182 expression) levels of miR-182. (**I and J**) The percentages of leukemic blasts in BM (**I**) and PB (**J**) were analyzed in AML patients with low expressions of miR-182 (below median) and high expressions of miR-182 (above median). *P < 0.05; **P < 0.01; ***P < 0.001.

To investigate the expression of miR-182 in AML blasts, we first measured the levels of miR-182 in BM mononuclear cells from 96 untreated AML blasts (Table S1) and 21 healthy volunteers for normal controls (NCs). The average of miR-182 expression was substantially lower in AML patients compared with NCs (Figure 1A). Furthermore, miR-182 expressions was subtype-dependent, which was lower in AML patients with abnormal karyotype t(8;21) and t(11q23) but not with t(15;17) and inv(16) than NCs (Figure 1B). However, no significant differences in miR-182 expressions were found between t(8;21), t(11q23), t(15;17), and inv(16) (Figure 1C). In addition, miR-182 expressions were lower in FAB subtypes M1, M2, M4, and M5, but not in M3 compared with NCs (Figure 1D). Also, there were no significant differences in miR-182 expressions between M1, M2, M3, M4, and M5 (Figure 1E). To further analyze the expressions of miR-182 in various different subpopulation of leukemic cells, we isolated BM CD34^+^ progenitor cells as undifferentiated cells and CD34^-^ differentiated cells from seven AML samples. miR-182 expressions were lower in CD34^+^ cells than CD34^-^ cells in 6 of 7 (85.7%) samples (Figure 1F).

We then explored whether miR-182 expression was associated with clinical outcome in our adult AML cohort (2015-2020). AML patients with higher level of miR-182 (above the median) are associated with better five-year overall survival (OS, Figure 1G, HR = 1.97; 95% CI = 1.13-3.45; *P* = 0.011) and five-year disease-free survival (DFS, Figure 1H, HR = 1.85; 95% CI = 1.06-3.22; *P* = 0.02) in comparison to those with lower level of miR-182 (below the median).

Consistently, AML patients with higher level of miR-182 showed decreased percentage of blasts in BM (Figure 1I) and peripheral blood (PB, Figure 1J), suggesting a correlation among the high miR-182 expression, the low percentage of AML blast, and the extended overall survival time. However, miR-182 expression was independent of age, gender, and molecular genetic abnormality (Table S1).

### miR-182 functions as tumor suppressor miRNA in human leukemic cells

**Figure 2:**
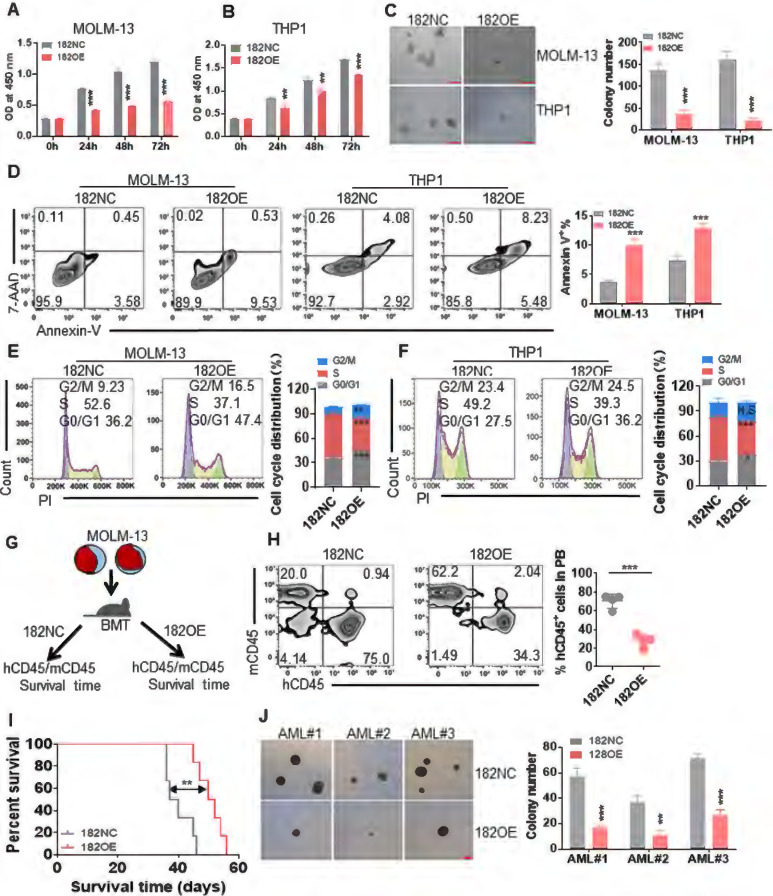
** Anti-leukemogenesis of miR-182 in human leukemic blasts *in vitro* and *in vivo*.** MOLM-13 and THP1 cells were transduced with MSCV vector overexpressing miR-182 (182OE) or negative control MSCV-NC (182NC). (**A and B**) CCK8 was measured in different times in MOLM-13 and THP1 cells overexpressing miR-182 or NC. (**C**) MOLM-13 and THP1 cells overexpressing miR-182 or NC (2×10^3^/dish) were plated in methylcellulose medium for ten days to count colony number. Bar scales represent 200 μm. (**D**) Apoptosis was measured in MOLM-13 and THP1 cells overexpressing miR-182 or NC by Annexin V and 7-AAD staining. (**E and F**) Cell cycle distribution in MOLM-13 and THP1 cells, which were transduced with 182OE or 182NC. Shown are the representative plots (left) and statistical analysis of G2/M, S, and G0/G1 phase. (**G**) An illustration of the MOLM-13-transplanted NSG mice model. MOLM-13 cells were transduced with 182OE or 182NC and xenografted in NSG mice. The percentage of human CD45 (hCD45)/murine CD45 (mCD45) was measured as chimerism, and OS time was analyzed. (**H**) hCD45/mCD45 was measured in PB from MOLM-13-182OE and MOLM-13-182NC-xenografted NSG mice. Shown are the representative plots (left) and statistical analysis of the percentage of hCD45/hCD45+mCD45 cells (right). (**I**) OS was determined in MOLM-13-182OE or MOLM-13-182NC-xenografted NSG mice. (**J**) Colony numbers were counted in three CD34^+^ primary AML blasts transduced with 182OE or 182NC. AML blasts (1×10^3^/dish) were plated in methylcellulose medium for ten days to count colony numbers. Bar scales represent 200 μm. *P < 0.05; **P < 0.01; ***P < 0.001 versus 182NC. Using log-rank test for survival time.

To identify the role of miR-182 in leukemic cells *in vitro*, leukemia cell lines (MOLM-13 and THP1) harboring MLL rearrangements, were transduced with retrovirus vector MSCV-miR-182 (182OE) or negative control vector MSCV-NC (182NC). qRT-PCR indicated the successful overexpression of miR-182 in leukemic cells (Figure S1A). Then, cell proliferation, colony formation, apoptosis, and cell cycle were measured in MOLM-13 and THP1 cells transduced with 182OE or 182NC. Cell proliferation indicated by OD450 was significantly reduced in leukemic cells transduced with 182OE compared with 182NC (Figure 2A-B). Furthermore, overexpression of miR-182 reduced about 70-80% of colonies in leukemic cells compared with 182NC (Figure 2C). In addition, overexpression of miR-182 slightly induced apoptosis (Figure 2D), but did not affect cell differentiation (Figure S1B-C). Also, miR-182 overexpression increased the percentage of AML cells into G0/G1 phases, while decreased the percentage of cells in the S phase (Figure 2E-F), suggesting that miR-182 could block the cell cycle.

Because MOLM-13 and THP1 are MLL-rearranged cells, we then explored the anti-leukemic effects of miR-182 on K562 and OCI-AML3 cells, which are non-MLL-rearranged cells. qRT-PCR indicated the successful overexpression of miR-182 in K562 and OCI-AML3 cells (Figure S1D). Overexpression of miR-182 reduced cell proliferation in K562 (Figure S1E) and OCI-AML3 cells (Figure S1F). Also, overexpression of miR-182 induced apoptosis in K562 (Figure S1G) and OCI-AML3 cells (Figure S1H). These data preliminarily indicated that miR-182 inhibited leukemia cell growth and proliferation *in vitro*.

To further assess the anti-leukemic effects of miR-182 *in vivo*, we transplanted MOLM-13 with 182OE and 182NC into NSG mice (Figure 2G). The chimerism represented as human CD45 (hCD45)/murine CD45 (mCD45) plus hCD45 was measured in PB when the mice showed signs of illness. The chimerism was decreased by 3.0-fold in PB from MOLM-13 (182OE)-transplanted NSG mice compared with MOLM-13 (182NC)-transplanted mice (Figure 2H). Finally, overexpression of miR-182 extended the OS time in comparison to 182NC (Figure 2I).

Moreover, we performed colony forming assay with primary CD34^+^ AML blasts which were isolated and transduced with 182OE or 182NC (n = 3). Overexpression of miR-182 decreased colony numbers in all three AML blasts compared with 182NC (Figure 2J). However, overexpression of miR-182 did not affect the colonies in normal CD34^+^ HSPC (n = 3, Figure S1I).

### Depletion of miR-182 accelerates AML progression by increasing the self-renewal of LSC in MLL-AF9-transformed murine AML model

Since overexpression of miR-182 inhibited proliferation of leukemic cells but not normal HSPCs, we performed loss of function studies with a miR-182^-/-^ (182KO) mice model 37, 38 to further explore miR-182 function during leukemogenesis using MLL-AF9-transformed AML model 4. PCR indicated a successful genotype of 182KO and 182WT mice (Figure S2A). BM and spleen cells were isolated from 182WT and 182KO mice and miR-182 expression was measured. We found that miR-182 level was almost completely depleted in BM and spleen cells from 182KO mice compared with 182WT mice (Figure S2B). To explore whether only miR-182 was depleted, miR-183 and miR-96 expressions were measured, because miR-183 and miR-96 locate near the miR-182 and these three miRNAs form a cluster 39. miR-183 and miR-96 expressions were not depleted in BM cells from 182KO mice compared with 182WT mice (Figure S2C-D). BM Lin^-^ cells were isolated from 182WT and 182KO mice and transduced with MLL-AF9 fusion gene, followed by transplantation in lethally irradiated mice (Figure S2E). BM GFP^+^ cells representing leukemic blasts were isolated from 182WT and 182KO AML mice, and qRT-PCR analysis indicated that miR-182 had been deleted efficiently in 182KO AML mice compared with 182WT AML mice (Figure S2F).

**Figure 3:**
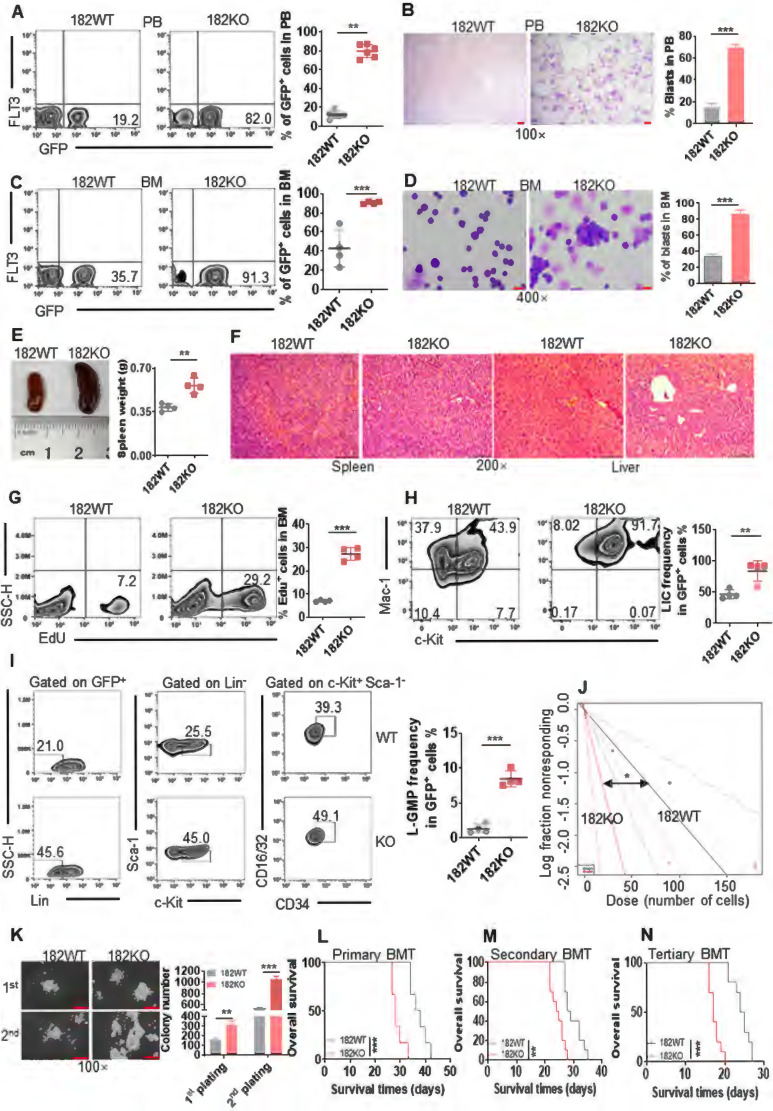
** Depletion of miR-182 (182KO) accelerates the development of MLL-AF9-transformed murine AML than wild-type control (182WT).** (**A and C**) The percentages of GFP^+^ cells were measured in PB (**A**) and BM (**C**) in 182WT (n = 4) AML cells compared with 182KO (n = 4). Shown are the representative plots (left) and statistical analysis of GFP^+^ cells (right). (**B and D**) The representative images of PB (**B**) and BM (**D**) smears by Wright-Giemsa stain in 182WT compared with 182KO AML cells (left) and statistical analysis of leukemic blasts (right). Bar scales represent 10 μm for PB and 20 μm for BM. (**E**) The representative images of the spleen (left) and statistical analysis of spleen weight (right). n = 4 for 182WT and 182KO AML cells, respectively. (**F**) The representative images of HE stain of spleen and liver tissues. Bar scales represent 100 μm for spleen and liver tissues. (**G**) 182WT (n = 4) and 182KO (n = 4) AML blasts were incubated with EdU for 2 h *in vitro*, and EdU^+^ cells were measured in GFP^+^ cells by flow cytometry. Shown are the representative flow cytometry plots (left) and statistical analysis of EdU^+^ cells (right). (**H**) The frequencies of LIC (c-Kit^+^Mac-1^+^) were measured in BM 182WT (n = 4) and 182KO (n = 4) AML cells. Shown are the representative flow cytometry plots (left) and statistical analysis of LIC (right). (**I**) The frequencies of BM L-GMP (Lin^-^c-Kit^+^Sca-1^-^CD34^+^CD16/32^+^) were measured in BM 182WT (n = 4) and 182KO (n = 4) AML cells. Shown are the representative flow cytometry plots (left) and statistical analysis of L-GMP cells (right). (**J**) Limiting dilution assay of BM GFP^+^ cells from secondary BMT leukemic mice with 182WT (n = 6) and 182KO (n = 6). The frequency of LSC and *P*-value were counted by L-calc software. (**K**) GFP^+^ 182WT and 182KO murine AML blasts (2×10^3^/dish) were plated in methylcellulose medium for ten days for first and secondary plating. Shown are the representative pictures (left) and statistical analysis of colony (n = 3). Bar scales represent 20 μm. (**L-N**) Kaplan-Meier curves indicating the effects of miR-182 knockout on MLL-AF9-induced primary (L, n = 6), secondary (M, n = 10), and tertiary BMT (N, n = 10). *P < 0.05; **P < 0.01; ***P < 0.001. Using log-rank test for survival time.

GFP^+^ cells were first measured in PB. A substantially increased percentage of GFP^+^ cells was observed in PB from 182KO AML mice than 182WT AML mice (Figure 3A). Also, the Wright-Giemsa stain indicated that depletion of miR-182 dramatically increased the leukemic blasts in PB from 182KO AML mice compared to 182WT AML mice (Figure 3B). Furthermore, knockout of miR-182 increased about 4-fold of leukemic blasts by measuring GFP^+^ cells (Figure 3C) and Wright-Giemsa stain (Figure 3D) in BM cells. Also, GFP^+^ cells were substantially increased in the spleen from 182KO AML mice compared to 182WT control (Figure S2G). To determine whether depletion of miR-182 enhanced the infiltration of leukemic blasts in the spleen, we measured the weight of spleen in 182WT and 182KO AML mice. The weight of the spleen increased about 50% in 182KO AML mice compared with 182WT AML mice (Figure 3E). Meanwhile, depletion of miR-182 substantially enhanced the infiltration of leukemic blasts in the spleen and liver in 182KO AML mice compared with 182WT AML mice (Figure 3F). To assess the proliferation status of leukemic cells, Edu staining was performed in BM cells. Edu^+^ cells were almost 3-fold higher in BM GFP^+^ blasts from 182KO AML mice than 182WT AML mice (Figure 3G). These results revealed that depletion of miR-182 increases leukemic cell proliferation and facilitates AML development.

To further explore whether the depletion of miR-182 enhanced the self-renewal capacity of LSC, BM GFP^+^ cells were sorted from primary 182WT and 182KO AML mice and transplanted into recipient mice as secondary BMT. We first measured immunophenotypic leukemia-initiating cell (LIC, c-Kit^+^/Mac-1^+^), a marker for leukemia progenitor cell. LIC was increased by above 2-fold in 182KO AML mice compared with 182WT AML mice (Figure 3H). Next, L-GMP (Lin^-^c-Kit^+^Sca-1^-^CD34^+^/CD16/32^+^) cells, widely considered as LSC 4, 40 in MLL-AF9-induced AML model, were measured in secondary BMT mice. LSC frequencies were increased by about 4-fold in BM (Figure 3I) and spleen GFP^+^ cells (Figure S2H) from 182KO AML mice compared with 182WT AML mice. Then, we used limiting dilution analysis (LDA) to assess functional LSC 36. The estimated frequency of LSC in 182KO AML mice was increased by about 3-fold than that in 182WT AML mice by LDA (Figure 3J and Table S3). Next, GFP^+^ cells were sorted for colony formation assay. First and secondary plating also demonstrated that colony number was increased in 182KO AML blasts compared with 182WT blasts (Figure 3K). Finally, we performed serial BMT with the same number of GFP^+^ blasts to evaluate miR-182 knockout in the self-renewal ability of LSC. Depletion of miR-182 substantially reduced the OS time in primary (Figure 3L), secondary (Figure 3M), and tertiary BMT (Figure 3N), respectively. It confirmed that the deletion of miR-182 enhanced the self-renewal capacity of LSC/LIC, accelerating AML progression.

Furthermore, another AML1-ETO9a (A/E9a)-induced M2 AML model was used to evaluate the function of miR-182 in certain types of leukemia 41. Recipient mice receiving miR-182-depleted A/E9a^+^ AML blasts had markedly shortened OS time during primary and secondary BMT than those receiving miR-182WT A/E9a^+^ AML blasts (Figure S2I-J). It indicated that the low expression of miR-182 observed in M2 subtypes was also relating to AML progression.

To test whether the homing ability of miR-182-null leukemia cells affects the survival of AML mice, a total of 2×10^6^ GFP^+^ blasts isolated from 182WT and 182KO AML mice were injected into the lethally irradiated mice, followed by measuring the homed cells (GFP^+^) in BM at 16 h after transplantation. We found no significant difference in GFP^+^ cells between 182WT and 182KO AML mice (Figure S2K). We then transplanted the same number of GFP^+^ blasts from MLL-AF9-transformed AML mice into normal 182WT and 182KO mice, followed by counting OS time. No significant difference in OS was found between 182WT and 182KO mice (Figure S2L). These results indicated that homing ability of miR-182-null leukemia cells do not affect the progression of AML mice.

### miR-182 knockout in MLL-AF9-transformed leukemic blasts accelerates oncogenesis signal pathway

**Figure 4:**
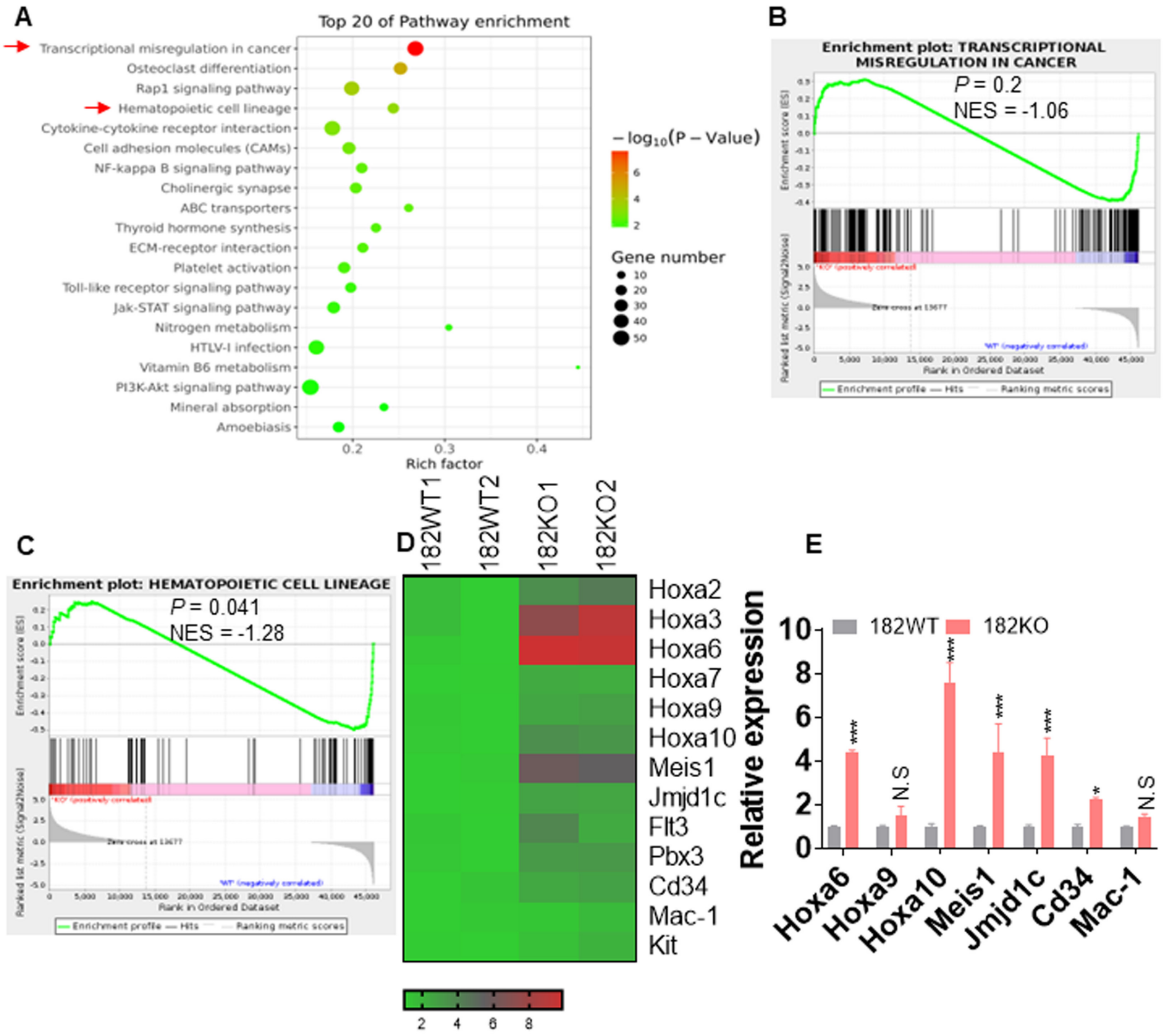
** Depletion of miR-182 facilitates the oncogenesis signal pathway.** BM GFP^+^ cells were isolated from 182WT and 182KO AML mice, and total RNA was extracted for RNA-seq. (**A**) GO (biological process) analysis of the RNA-seq data. (**B and C**) Gene Set Enrichment Analysis (GSEA) indicated significantly enriched higher (upper panel) or lower (lower panel) expression of genes in 182WT compared with 182KO AML cells. (**D**) Heat map showing expression of upregulated and downregulated genes in 182WT and 182KO AML cells. (**E**) qRT-PCR was performed in selected genes, which were enriched in 182KO (n = 3) compared with 182WT AML blasts (n = 3). *P < 0.05; **P < 0.01; ***P < 0.001. N.S: not significant.

To explore the underlying mechanisms that control the proliferation and self-renewal of LSC in miR-182-depleted murine AML blasts, BM GFP^+^ cells were sorted from 182WT and 182KO AML mice and performed for RNA-seq. Gene Ontology (GO, Figure 4A) and Gene Set Enrichment Analysis (GSEA, Figure 4B-C) demonstrated that signal pathways including transcriptional misregulation in cancer and hematopoietic cell lineage were enriched in 182KO AML cells in comparison to 182WT AML cells, although the *P* value in GSEA of transcriptional misregulation in cancer is not significant. Multiple enriched genes (*Hoxa6*, *Hoxa10*, and *Meis1*) in the pathway of transcriptional misregulation in cancer were associated with LSC self-renewal and survival (Figure 4D). Also, *Cd34* and *Mac-1* in the pathway of hematopoietic cell lineage were associated with undifferentiation status of leukemic cells. We then validated its expression by qRT-PCR and found that these genes were indeed increased in 182KO compared with 182WT AML blasts (Figure 4E). Therefore, the results from RNA-seq were consistent with the phenotype from 182WT and 182KO AML mice that depletion of miR-182 accelerates AML progression through increasing the self-renewal of LSC.

### The overexpression of miR-182 could benefit AML treatment

**Figure 5:**
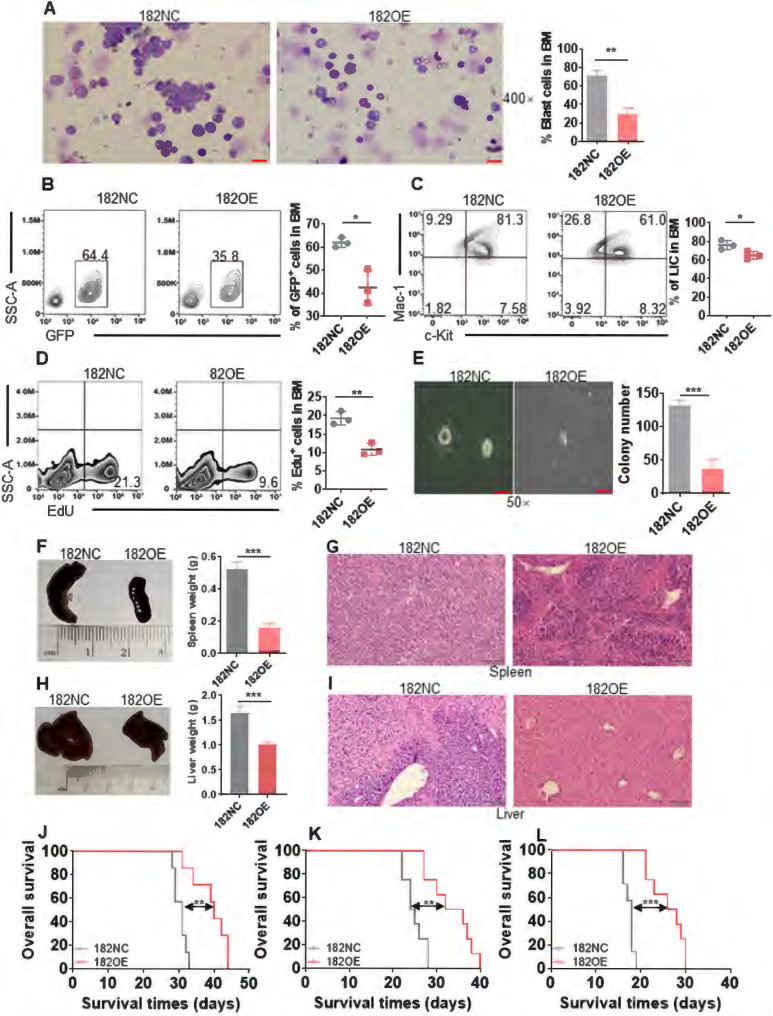
** Overexpression of murine miR-182 (182OE) weakens the development of MLL-AF9-transformed murine AML.** (**A**) The representative images of BM smears by Wright-Giemsa stain in 182NC compared with 182OE AML cells (left) and statistical analysis of leukemic blasts (right). Bar scales represent 20 μm. (**B**) The percentages of BM GFP^+^ cells were determined in 182NC (n = 3) compared with 182OE AML cells (n = 3). Shown are the representative plots (left) and statistical analysis of GFP^+^ cells (right). (**C**) The percentages of BM LIC were determined in 182NC (n = 3) compared with 182OE (n = 3) AML cells. Shown are the representative plots (left) and statistical analysis of LICs (right). (**D**) 182NC (n = 3) and 182OE (n = 3) AML blasts were incubated with buffer containing EdU *in vitro,* and the incorporation of EdU was measured by flow cytometry. Shown are the representative plots (left) and statistical analysis of EdU^+^ in BM GFP^+^ cells (right). (**E**) BM GFP^+^ cells (2×10^3^/dish) from 182NC and 182OE AML mice were sorted for colony formation assay. Shown are the representative pictures (left) and statistical analysis of colony number (right). Bar scales represent 40 μm. (**F and H**) The representative images of the spleen (left, **F**) and liver (left, **H**) and statistical analysis of spleen weight (right, **F**) and liver weight (right, **H**). (**G and I**) HE stain for spleen (**G**) and liver (**I**) tissues in recipients receiving 182NC AML cells compared with those receiving 182OE AML cells at the endpoint. Bar scales represent 100 μm. (**J-L**) Kaplan-Meier curves and *P* values (log-rank test) from primary (**J**), secondary (**K**), and tertiary BMT (**L**) are shown, respectively. *P < 0.05; **P < 0.01; ***P < 0.001. Using log-rank test for survival time.

To further identify the function of miR-182 and explore its potential application in AML clinical treatment, MLL-AF9^+^ AML cells were transduced with LVX-puro-mmu-miR-182 (182OE) or negative vector (182NC). As indicated in Figure S3A, miR-182 expression was increased by 5-fold in 182OE-AML cells than 182NC AML cells. Wright-Giemsa stain indicated that leukemic blasts dramatically decreased in PB (Figure S3B) and BM (Figure 5A) from recipient mice receiving 182OE than those receiving 182NC AML cells. GFP^+^ cells were significantly decreased in PB (Figure S3C) and BM (Figure 5B) from recipient mice receiving 182OE compared with 182NC AML cells. Also, overexpression of miR-182 markedly decreased the percentage of LIC (Figure 5C) and Edu-positive cells (Figure 5D), as well as the colony number (Figure 5E). In addition, 182OE substantially decreased the weight of the spleen (Figure 5F) and liver (Figure 5H), due to the inhibition of the infiltration of leukemic blasts in the spleen (Figure 5G) and liver (Figure 5I), which is opposite to the results of miR-182 deletion. Finally, serial BMT using 182NC and 182OE AML cells were performed. OS time in primary BMT was significantly extended in recipients receiving 182OE compared with 182NC AML cells (Figure 5J, median survival, 40 days vs 31 days, *P* < 0.01), secondary BMT (Figure 5K, median survival, 34 days vs 24.5 days, *P* < 0.01) as well as tertiary BMT (Figure 5L, median survival, 27 days vs 18 days, *P* < 0.001). All these data indicated that miR-182 overexpression inhibits leukemogenesis *in vivo* by decreasing AML self-renew ability and proliferation, which might benefit AML treatment.

### miR-182 is dispensable for normal hematopoiesis

**Figure 6:**
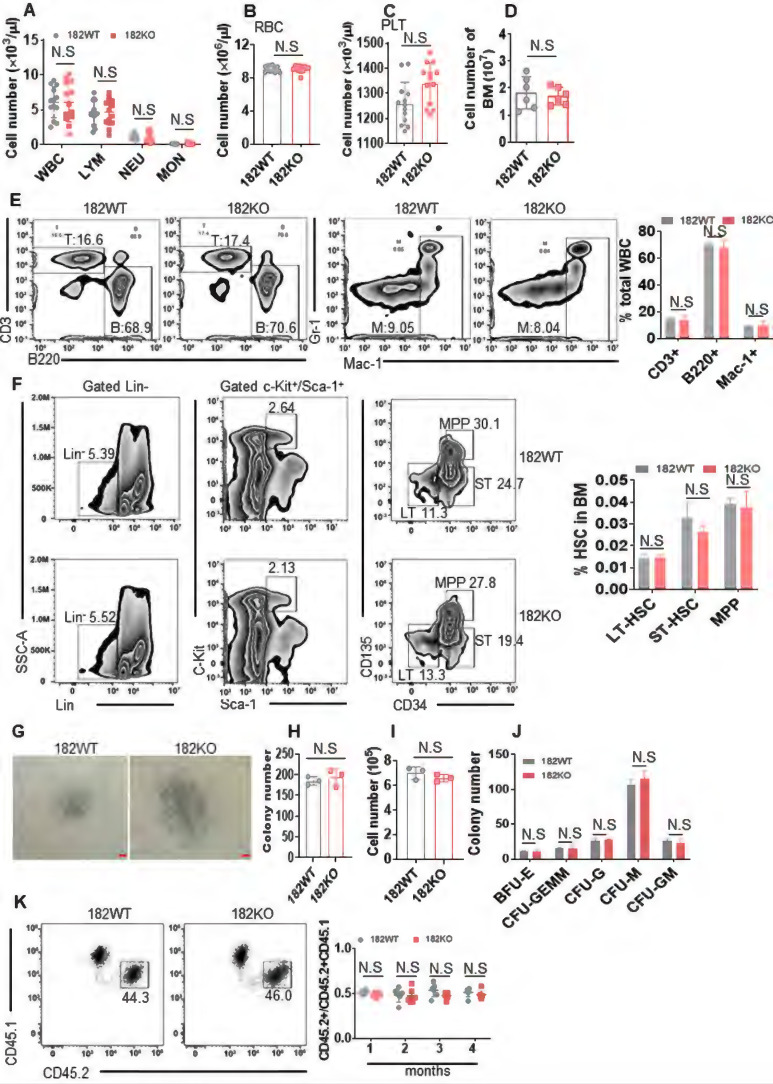
** miR-182 is not required for the normal hematopoiesis.** (**A-C**) The total numbers of WBC, LYM, NEU, MON, RBC, and PLT were counted by automatic blood cell counter in 182WT (n = 17) and 182KO (n = 16) mice. (**D**) The total numbers of BM mononuclear cells were counted in 182WT (n = 6) and 182KO (n = 6) mice. (**E**) T (CD3^+^), B (B220^+^), and myeloid cells (Mac-1^+^, Gr-1^+^) frequencies were performed by flow cytometry in PB from 182WT (n = 4) versus 182KO (n = 4) mice. (**F**) The percentages of long-term hematopoietic stem cell (LT-HSC, Lin^-^c-Kit^+^Sca-1^+^CD34^-^CD135^-^), short-term hematopoietic stem cell (ST-HSC, Lin^-^c-Kit^+^Sca-1^+^CD34^+^CD135^-^), and multipotential progenitor (MPP, Lin^-^c-Kit^+^Sca-1^+^CD34^+^CD135^+^) were measured by flow cytometry in 182WT (n = 4) and 182KO (n = 4) mice. (G-J) BM LSK (Lin^-^c-Kit^+^Sca-1^+^) cells were sorted from 182WT (n = 3) and 182KO (n = 3) mice and plated on methylcellulose medium for colony number. Shown are the representative pictures (**G**) and statistical analysis of colony number (**H**), total number (**I**), and the classification of burst-forming unit-erythroid (BFU-E), colony-forming unit-granulocyte, erythroid, macrophage, and megakaryocyte (CFU-GEMM), colony-forming unit-granulocyte (CFU-G), colony-forming unit-macrophage (CFU-M), and colony-forming unit-granulocyte and macrophage (CFU-GM) (**J**). Bar scales represent 100 μm. (**K**) *In vivo* competition assays for CD45.2/CD45.2+CD45.1 after transplantation for the indicated months. Shown are the representative plots (left) and statistical analysis of CD45.2/CD45.2+CD45.1 (right). N.S: not significant.

Since miRNAs may affect both normal and malignant hematopoietic function, it is hard to develop miRNA as a suitable target in AML 14. To clarify the role of miR-182 in normal hematopoiesis, we performed blood cell count with miR-182 KO (182KO) and miR-182 WT (128WT) mice. White blood cell (WBC), lymphocyte (LYM), neutrophil (NEU), monocytes (MON), red blood cell (RBC), and platelet (PLT) counts were comparable between 182WT and 182KO mice at 3-month-old mice, respectively (Figure 6A-C). Furthermore, the total BM cell count was similar between 182WT and 182KO mice (Figure 6D). Also, the percentages of T (CD3^+^), B (B220^+^), and myeloid cells (Mac-1^+^, Gr-1^+^) were comparable in PB from 182WT mice versus 182KO mice (Figure 6E). It suggested that miR-182 deletion might not affect normal hematopoietic pool.

After that, the frequencies of mouse LT-HSC (Lin^-^c-Kit^+^Sca-1^+^CD34^-^CD135^-^), ST-HSC (Lin^-^c-Kit^+^Sca-1^+^CD34^+^CD135^-^), and MPP (Lin^-^c-Kit^+^Sca-1^+^CD34^+^CD135^+^) were measured in BM cells. There were no significant differences in the frequencies of LT-HSC, ST-HSC, and MMP between 182WT and 182KO mice (Figure 6F). To clarify if miR-182 deletion affected the self-renewal ability of HSPC, colony assay was performed by using LSK (Lin^-^c-Kit^+^Sca-1^+^) cells, which were sorted from 182WT and 182KO mice. The colony number and total number of LSK cells were comparable in 182WT mice versus 182KO mice (Figure 6G-I). Also, the numbers of BFU-E, CFU-GEMM, CFU-M, CFU-GM, and BFU-E colonies in 182WT mice were indistinguishable from 182KO mice, respectively (Figure 6J). Finally, we performed a competitive transplantation to evaluate the effects of miR-182 depletion on the self-renewal ability. Knockout of miR-182 did not affect the percentage of CD45.2/(CD45.2+CD45.1) in recipient mice (Figure 6K). Taken together, these results suggest that depletion of miR-182 facilitates the self-renewal activity of LSC, but miR-182 unlikely could affect the normal hematopoiesis and the function of HSPC.

### miR-182 functions as a tumor suppressor by directly targeting *BCL2* and* HOXA9*

**Figure 7:**
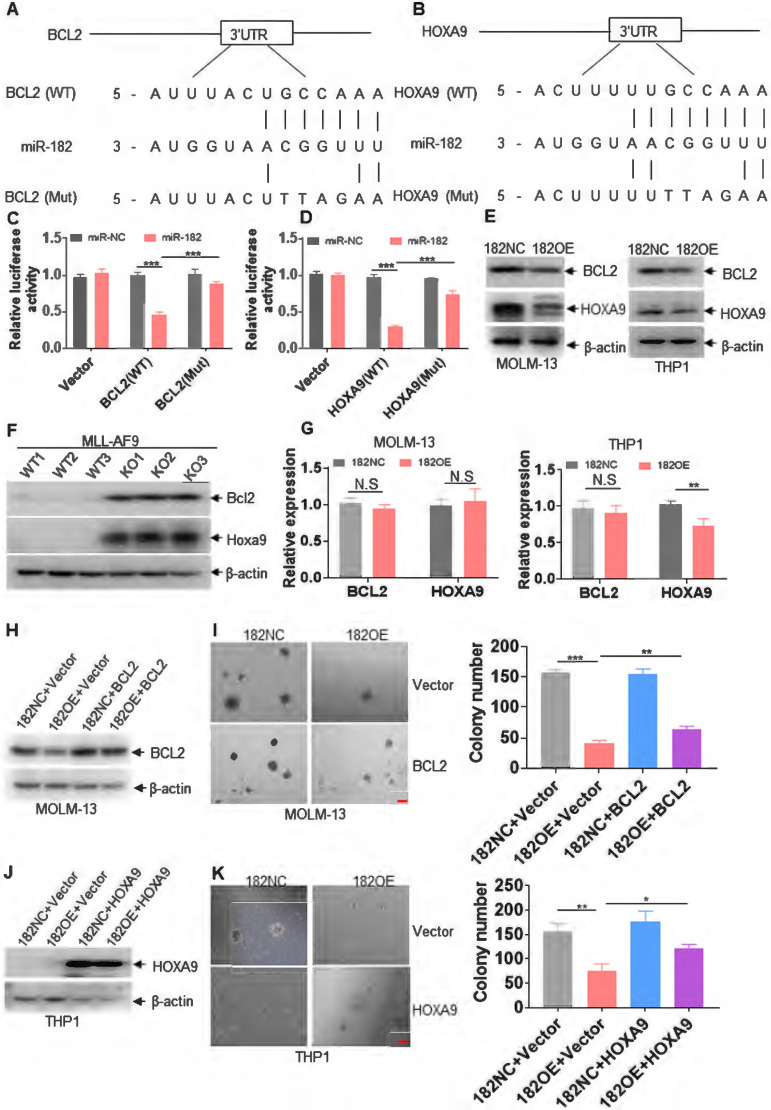
** miR-182 directly targets *BCL2* and* HOXA9*.** (**A and B**) Schematic of putative binding sites for miR-182 in 3'-UTR of *BCL2* and *HOXA9*. (**C and D**) 293T cells were transfected with wild-type psicheck-2-BCL2-3'-UTR (WT), psicheck-2-BCL2-3'-UTR (Mut), psicheck-2-HOXA9-3'-UTR (WT), and psicheck-2-HOXA9-3'-UTR (Mut) for 24 h, following by the transfection with miR-182 mimic or scramble (miR-NC) for another 24 h. Firefly and Renilla luciferase activities were both measured. Histograms indicated that the Firefly luciferase activities were normalized to Renilla luciferase activities. (**E**) The protein expressions of BCL2 and HOXA9 were measured in MOLM-13 and THP1 cells transduced with 182OE or 182NC. (**F**) The protein levels of Bcl2 and Hoxa9 were measured in MLL-AF9-transformed murine AML blasts with 182WT or 182KO. (**G**) The transcript expressions of *BCL2* and *HOXA9* were measured in MOLM-13 and THP1 cells transduced with 182OE or 182NC. (**H and J**) The protein expressions of BCL2 and HOXA9 were determined in 182OE-and 182NC-transduced MOLM-13 and THP1 cells, following by transduction with LVX-HOXA9 or LVX-NC (Vector), respectively. (**I and K**) Colonies were counted in 182OE- and 182NC-transduced MOLM-13 and THP1 cells, which were transduced with LVX-HOXA9 or LVX-NC (Vector), respectively. Shown are the representative pictures (left) and statistical analysis of the colony (right). Bar scales represent 200 μm. *P < 0.05; **P < 0.01; ***P < 0.001. N.S: not significant.

To further select the target genes by miR-182, software including TargetScan (http://targetscan.org) 31, 42 and the microRNA.org 43 was utilized to predict possible target genes. *BCL2* and* HOXA9*, which are associated with proliferation and survival, were finally selected for the following tests (Figure 7A-B). We then analyzed human and murine mature miR-182 sequences and found these two sequences are conserved (Figure S4A). Only one base is different between human and murine miR-182 sequences (Figure S4A). Also, the miR-182 target regions in 3'-UTR of *HOXA9* and* BCL2* are conserved for human and murine miR-182 (Figure S4B-C). To determine whether *BCL2* and* HOXA9* are directly targeted by miR-182, putative miR-182-binding sites were amplified and subcloned into vector psi-Check-2, and luciferase (Luc) activities were measured. Overexpression of miR-182 reduced the Luc activity of *BCL2* and* HOXA9* in comparison to negative control (Figure 7C-D). However, overexpression of miR-182 did not affect the Luc activity of psi-Check-2 carrying mutated miR-182-binding sites (Figure 7C-D). We then measured BCL2 and HOXA9 protein levels in 182OE- or 182NC-expressing leukemic cells and found that 182OE decreased BCL2 and HOXA9 protein expressions in MOLM-13 and THP1 cells compared with 182NC (Figure 7E). Also, overexpression of miR-182 reduced the protein expressions of BCL2 and HOXA9 in K562 and OCI-AML3 cells (Figure S4D-E). Furthermore, Bcl2 and Hoxa9 protein expressions were substantially increased in BM blasts from 182KO compared with 182WT AML mice (Figure 7F, exposure in very short time). In another blots with exposure for a relatively long time, MLL-AF9-positive cells show reasonable expressions of Bcl2 and Hoxa9 (Figure S4F). However, 182OE did not affect the transcriptional expression of *BCL2* in MOLM-13 and THP1 cells (Figure 7G), as well as *HOXA9* in MOLM-13 cells, but slightly decreased the transcriptional expression of *HOXA9* in THP1 cells (Figure 7G). It indicated that miR-182 mainly affects the post-transcription processing or translation of *BCL2* and *HOXA9*.

To explore whether *BCL2* and *HOXA9* overexpression can rescue miR-182-induced anti-leukemic property, colony formation was counted in 182OE- or 182NC-expressing leukemic cells, which were further transduced with LVX-based vector overexpressing *BCL2* or *HOXA9*, respectively. Overexpression of *BCL2* (Figure 7H-I) and *HOXA9* (Figure 7J-K) lacking 3'-UTR almost rescued the decreased protein expression and partially rescued the decreased colonies by miR-182, respectively. To avoid the overexposure of the blots for the overexpression of HOXA9, blots in Figure 7J were exposed for very short time. Therefore, the blots of THP1 control cells almost show no protein expression of HOXA9. When the blots of Figure 7J were exposed for a relatively long time, THP1 control cells expressed a reasonable level of HOXA9 (Figure S4G). However, *BCL2* and* HOXA9* overexpression inhibited miR-182-induced anti-leukemia activity to some extent, suggesting that there were other targets regulated by miR-182 in AML. In short, miR-182 binds 3'-UTR of *BCL2* and *HOXA9* to inhibit their protein expression.

### miR-182 expression is suppressed by promoter methylation

**Figure 8:**
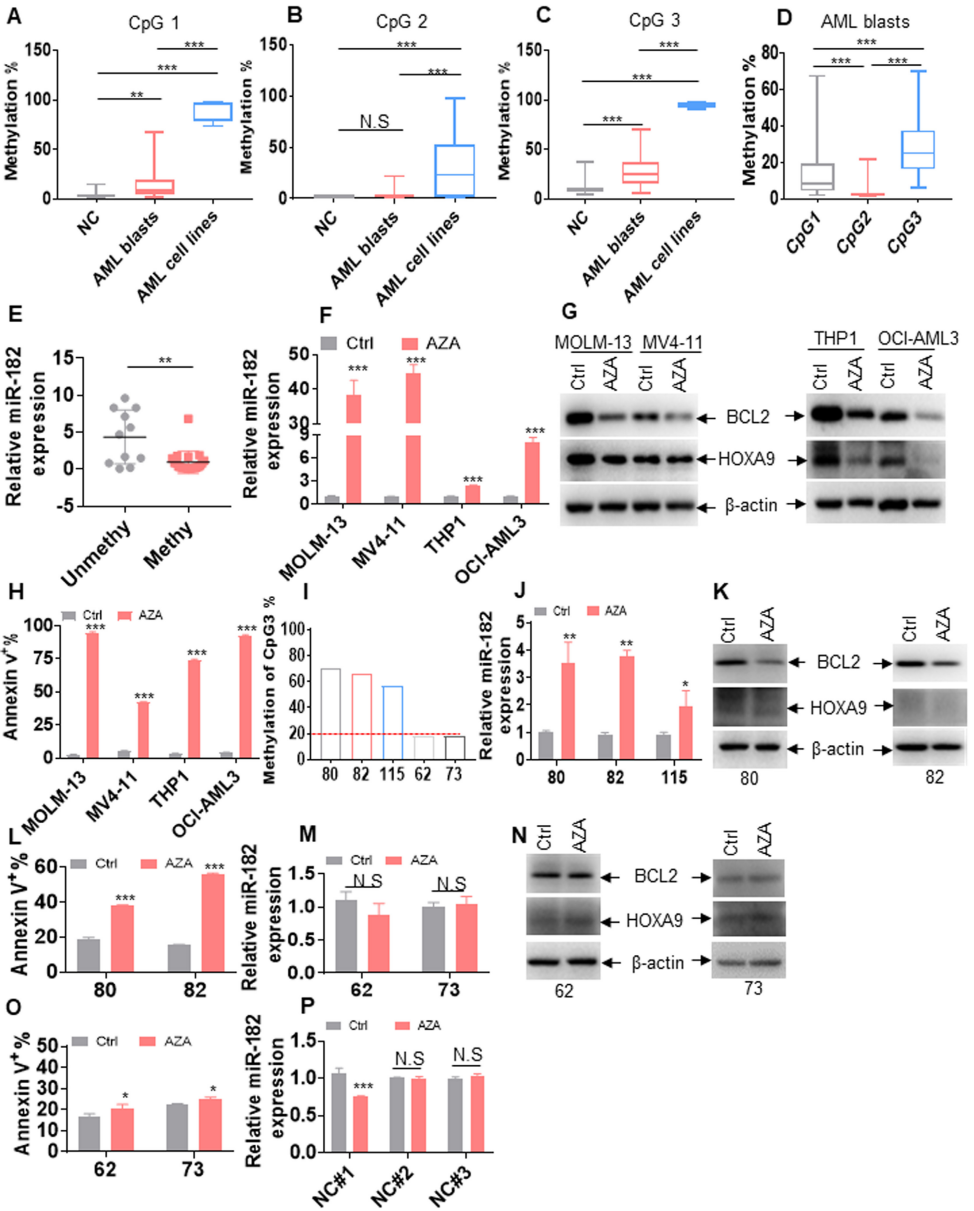
** miR-182 promoter is hypermethylated in leukemic cells.** (**A-C**) The percentages of DNA methylation of CpG islands 1 (**A**), 2 (**B**), and 3 (**C**) in normal control (NC), primary AML blasts, and AML cell lines. (**D**) The percentages of DNA methylation of CpG islands 1, 2, and 3 in primary AML blasts. (**E**) miR-182 expressions were measured in AML blasts with miR-182 methylation (> 20%) and unmethylation (< 20%). (**F**) miR-182 expressions were measured in the indicated leukemic cell lines treated with 5-azacytidine (5 μM, AZA) or not for four days. (**G**) BCL2 and HOXA9 protein levels were measured in the AZA-treated or-untreated leukemic cells for four days. (**H**) Apoptosis was measured in the AZA-treated or untreated leukemic cells for four days. (**I**) The percentage of DNA methylation of CpG island 3 in five AML patients. (**J**) miR-182 expression was measured in AML patients 80, 82, and 115, which were treated with AZA or not for four days. (**K**) BCL2 and HOXA9 protein levels were measured in AML patients 80 and 82, which were treated with AZA or not for four days. (**L**) Apoptosis was measured in AZA-treated or untreated AML patients 80 and 82. (**M**) miR-182 expressions were measured in AML patients 62 and 73, which were treated with AZA or not for four days. (**N**) BCL2 and HOXA9 protein levels were measured in AML patients 62 and 73 treated with AZA or not for four days. (**O**) Apoptosis was measured in AZA-treated or -untreated AML patients 62 and 73. (**P**) miR-182 expressions were measured in three NC samples treated with AZA or not for four days *P < 0.05; **P < 0.01; ***P < 0.001. N.S: not significant.

Since CpG island methylation-induced transcriptional silencing of tumor suppressor genes frequently occurs in cancer cells 17, we explored the possible CpG islands at pri-miR-182 promoter. Total of three CpG islands (CpG island 1-3) located 6-12 kb upstream of pri-miR-182 were found (Figure S5A-C). To determine whether DNA hypermethylation was present in leukemic blasts, we used MethylTarget^TM^ assays to measure methylation status in 8 AML cell lines, 34 untreated primary AML blasts, and 26 normal BM mononuclear cells as NCs. The average frequencies of CpG island 1 were higher in AML cell lines and primary AML blasts compared with NCs, respectively (Figure 8A, Figure S6). Also, the average frequencies of CpG island 3 were higher in AML cell lines (95.4%) and primary AML blasts (28.9%) in comparison to NCs (11.5%), respectively (Figure 8C, Figure S7). While, the average frequencies of CpG island 2 were similar in primary AML blasts than in NCs (Figure 8B, Figure S8-9). Furthermore, CpG island 3 and CpG island 1 were more heavily hypermethylated than CpG island 2 in primary AML blasts (Figure 8D), respectively. As expected, miR-182 expression was lower in AML patients with methylation (methylation% > 20%) than those with unmethylation (methylation% < 20%, Figure 8E and Table S4). These results demonstrated that CpG islands 3 and 1 but not CpG island 2 were hypermethylated in leukemic cells than NCs and miR-182 expression is likely suppressed by promoter methylation.

Next, we explored whether AZA restored the expression of miR-182. As expected, AZA markedly increased the expression of miR-182 in MOLM-13, MV4-11, THP1, and OCI-AML3 cells (Figure 8F), which all present miR-182 hypermethylation (Figure S7). In consistent with it, AZA treatment markedly decreased the protein levels of BCL2 and HOXA9 (Figure 8G) and substantially induced apoptosis in these four cell lines (Figure 8H, Figure S10A-B). Furthermore, we evaluated whether AZA increased the expression of miR-182 and presented anti-leukemic ability in primary AML blasts. Three primary AML blasts (80, 82, and 115) with hypermethylation (methylation% of CpG island 3 is above 40%, Figure 8I) and two AML blasts (62 and 73) with unmethylation (methylation% of CpG island 3 is below 20%, Figure 8I) were treated by AZA. As indicated in Figure 8J, AZA elevated the expression of miR-182 in primary AML80, 82, and 115, as well as substantially decreased BCL2 and HOXA9 expressions (Figure 8K) and induced apoptosis (Figure 8L, Figure S10C). However, AZA did not markedly increase miR-182 expression (Figure 8M) and decrease BCL2 and HOXA9 expressions (Figure 8N), as well as did not substantially induce apoptosis (Figure 8O, Figure S10D) in AML62 and 73. Finally, miR-182 expression was measured in three normal HSPCs (NC1-3) with unmethylation at pri-miR-182 promoter (Figure S7), which were treated with or without AZA. AZA treatment did not increase the expression of miR-182 in normal HSPCs (Figure 8P). These results demonstrated that AZA presents better anti-leukemic ability in AML blasts with hypermethylation at pri-miR-182 promoter compared with those with unmethylation.

### miR-182 promoter methylation determines the sensitivity of BCL2 inhibitor venetoclax (Ven)

**Figure 9:**
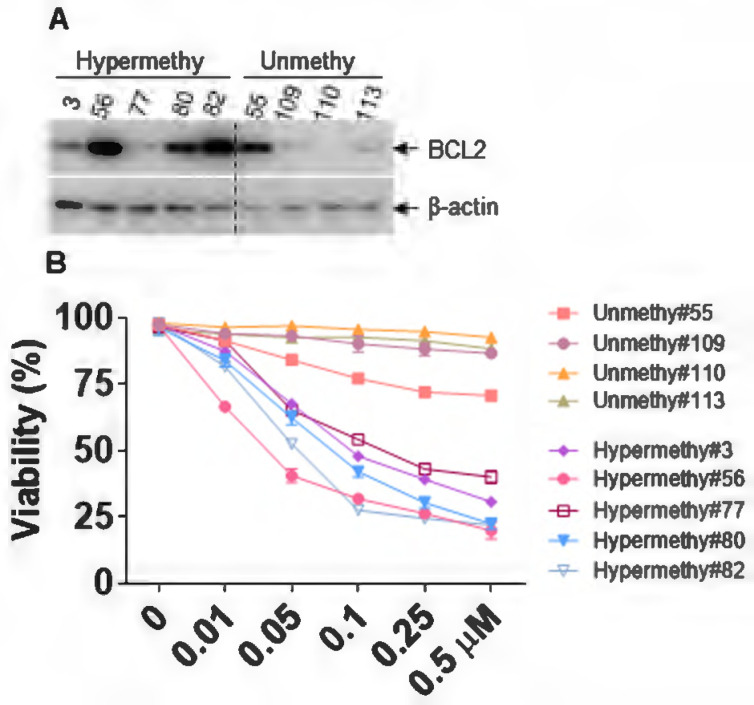
** miR-182 promoter methylation determines the sensitivity of Ven.** (**A**) The protein expressions of BCL2 were measured in AML blasts with miR-182 hypermethylation or unmethylation. (**B**) Cell viability was measured in AML blasts with miR-182 hypermethylation or unmethylation treated with different concentrations of Ven for 24 h *in vitro*.

Several studies have indicated that BCL2 expression strongly correlated with Ven sensitivity, and loss of BCL2 expression caused Ven resistance 44, 45. We first analyzed the protein expression of BCL2 in AML patients with miR-182 promoter methylation or unmethylation, which resulted in the low or high expression of miR-182, respectively (Figure 8E, Table S4). As indicated in Figure 9A, BCL2 protein expression was substantially higher in AML patients with miR-182 promoter hypermethylation compared with unmethylation. Furthermore, the sensitivity of Ven was determined *in vitro*. Our results show that AML patients with miR-182 promoter unmethylation are significantly more resistant for Ven treatment than those with miR-182 hypermethylation (Figure 9B), suggesting that miR-182 promoter methylation might determine the expression of BCL2 and the sensitivity of Ven treatment.

## Discussion

Our studies identified an important AZA-miR-182-BCL2/HOXA9 axis in AML blasts. However, AZA might regulate the expressions of BCL2/HOXA9 through other miRNAs or other genes. Also, more AML samples were needed for AZA treatment. miR-182 level was decreased in AML blasts whereby DNA hypermethylation at pri-miR-182 promoter was observed. Overexpression of miR-182 extended the OS of human AML cells-transplanted NSG mice and MLL-AF9-induced murine AML *in vivo*. Furthermore, depletion of miR-182 in MLL-AF9-transformed murine AML model facilitated the development of AML through increasing LIC/LSC frequency and function. Although previous reports indicated that miR-182 acts as oncogenic miRNA or tumor suppressor miRNA in different solid tumors 46, 47, our results confirm that miR-182 plays tumor suppressor role in AML cells. miRNAs have been reported to directly or indirectly regulate various targets with potential counteracting roles 35. Therefore, the cell context-dependent balance in miR-182-regulated targets might finally determine the biological roles in AML cells.

Our results demonstrated that depletion of miR-182 did not affect normal hematopoiesis. However, Wurm *et al*. reported that overexpression of miR-182 by lentivirus transduction in LSK cells decreased the percentage of mature granulocytes in BM and PB *in vivo*, but not developed a hyperproliferative disease or leukemia-like disease 29. This function might be due to the decreased expression of C/EBPα by the overexpression of miR-182 in murine LSK cells 29. However, considering that culture of lentivirus-transduced HSPCs *in vitro* will result in the differentiation, miR-182 knocking mouse model is still required to completely elucidate the effects of miR-182 on normal hematopoiesis.

HOXA9 is a master regulatory transcription factor to regulate self-renewal of LIC/LSC. HOXA9 overexpressing in LSC than HSPC is required for the MLL-AF9-transformed leukemia 48. Co-expressions of *Hoxa9* and *Meis1* in murine HSPC are sufficient to induce a fatal AML in xenografted mice 49. Furthermore, HOXA9 interacts with JMJD1C and PBX3 to maintain the phenotype and development in HOXA9-dependent and MLL-rearranged leukemia 5, 50. Therefore, these reports have indicated that *HOXA9* is the core gene for the proliferation and self-renewal LIC/LSC. However, until now no efficient therapeutic compounds directly inhibit or degrade HOXA9 protein expression. Thus, overexpression of miR-182 should be a promising tool to inhibit HOXA9 expression.

Recently, BCL2 has been reported to be overexpressed in ROS-low LSC and targeting BCL2 selectively eliminated LSC 51, demonstrating that BCL2 is an important target for LSC. Our results indicate that *BCL2* is a direct target for miR-182, which is consistent with other reports 52, 53. Therefore, restoring miR-182 expression by HMAs or miRNA mimics can decrease BCL2 expression. In addition to miR-182, *BCL2* was regulated by miR-15a/16-1 and low expression of miR-15a/16-1 led to the overexpression of BCL2 in chronic lymphocytic leukemia 54, 55. Also, *BCL2* mRNA stability was regulated by YBX1 in an m6^A^-dependent manner 56. Therefore, a complicated regulatory net contributes to the over expression of BCL2 in AML cells and targeting BCL2 protein by Ven substantially improves the overall survival in AML patients 57. However, Ven resistance which frequently occurs in partial AML patients with lost expression of BCL2, limits the further application of Ven 44.

Our results first demonstrated that the average miR-182 methylation is higher in AML patients than in NCs. Most AML patients presented miR-182 hypermethylation, while partial AML patients had miR-182 unmethylation. Furthermore, AML patients with miR-182 unmethylation had high expression of miR-182 followed by lost expression of BCL2 protein, which finally led to the resistance of Ven treatment *in vitro*. Therefore, miR-182 methylation might be a predicative marker for the sensitivity of Ven treatment. However, more AML samples with miR-182 unmethylation are required for the further tests *in vitro* and *in vivo*.

DNA methylation might be potential biomarker for tumor classification and predicative response to drug treatment because it has several advantages such as accuracy, stability, specificity, and easiness to be measured by MSP 58. Although multiple CpG islands at pri-miR-182 promoter were found in melanoma and renal cell carcinoma 46, 59, whether all these CpG islands are hypermethylated in leukemia cells are largely unknown. We first identified three CpG islands at the putative miR-182 promoter region, and found that CpG islands 3 and 1 are mostly hypermethylated than CpG island 2 in AML blasts. CpG island 2 is hypomethylated in both AML blasts and NCs. Furthermore, hypermethylation level of CpG island 3 is higher than CpG island 1 in AML blasts. Also, AZA increased miR-182 level in leukemic cells with hypermethylation at miR-182 promoter (CpG island 3>20%) and presented strong anti-leukemic activity by decreasing BCL2 and HOXA9 protein. While AZA did not upregulate miR-182 level in leukemic cells with unmethylation at miR-182 promoter and presented less anti-leukemic activity. Therefore, our studies identify a novel hypermethylation-miR-182-BCL2/HOXA9 axis in AML blasts and CpG island 3 methylation level might be a predicative biomarker for HMA drug treatment.

The marked characteristics of HSPC and LSC are the unique self-renewal ability. Although HSPC and LSC have common pathways to maintain self-renewal ability, self-renewal of LSC depends on some special genes, which are unnecessary for HSPC 60. Therefore, elucidating these independencies of HSPC versus LSC might provide more effective and less toxic therapies for AML treatment. Several reports have indicated that miRNAs could regulate HSPC and LSC self-renewal 13, 61. However, only a few studies have reported that miRNAs are required for LSC but not for HSPC self-renewal. Our results first demonstrated that miR-182 inhibited self-renewal in LSC but not in HSPC. miR-182 was dispensable for normal hematopoiesis, suggesting that miR-182 is an ideal target for LSC. These results might shed light on the different regulation of self-renewal in LSC and HSPC.

## Conclusions

Our studies concluded that miR-182 is a tumor-suppressor gene and inhibits the self-renewal of LSC but not HSPC via targeting *BCL2* and *HOXA9*, suggesting that miR-182 is potential therapy target for LSC. miR-182 expression is suppressed or silenced by promoter methylation, which then determines the sensitivity of Ven and provides a predictive biomarker for Ven in AML.

## Supplementary Material

Supplementary materials and methods, figures and tables.

## Abbreviations

miRNAs: microRNAs
AML: acute myeloid leukemia
LSCs: leukemia stem cells
HSPCs: normal hematopoietic stem progenitor cells
Ven: venetoclax
bone marrow transplantation: BMT
OS: overall survival
MLL: mixed-lineage leukemia
LIC: leukemia-initiating cell
miR-182: MicroRNA-182-5p
HMA: hypomethylation agent
AZA: 5-Azacytidine
WBC: white blood cell
LYM: lymphocyte
NEU: neutrophil
MON: monocytes
RBC: red blood cell
PLT: platelet
