# Supplementary material for: DNA hypermethylation-induced miR-182 silence targets BCL2 and HOXA9 to facilitate the self-renewal of leukemia stem cell, accelerate acute myeloid leukemia progression, and determine the sensitivity of BCL2 inhibitor venetoclax
Source: Theranostics. 2023 Jan 1;13(1):77–94. doi: 10.7150/thno.77404 (PMC9800726; doi:10.7150/thno.77404)
Supplement: Supplementary file 1 — Supplementary materials and methods, figures and tables. [file thnov13p0077s1.pdf]

Supplemental files

Supplemental material and methods

Chemical drugs

5-Azacitidine (AZA, Sigma-Aldrich, St. Louis, MO, USA), BCL-2 inhibitor venetoclax (Sigma-Aldrich), and puromycin (puro, Sigma-Aldrich) were dissolved in dimethyl sulphoxide and kept at -20℃ until use.

Western blot

Western blot analysis was performed using standard techniques. Briefly, leukemic cells were harvested and lysed by 1×SDS lysis buffer with phenylmethylsulfonyl fluoride, sodium orthovanadate, and protease inhibitors (Thermo Scientific, Waltham, MA, USA). Extracted protein was heated at 100℃ for 5 min, followed by rapid cooling at 4℃ for 5 min. Protein concentration was measured by a BCA assay (Thermo Scientific). Proteins (20 μg/well) were fractionated by electrophoresis through polyacrylamide gels (Bio-Rad, Richmond, CA, USA) and transferred to PVDF membranes. After incubation with TBS with 5% skim milk powder for 2 h, blots were incubated with primary antibodies over night at 4 °C and incubated with a secondary HRP-conjugated antibody for 1 h. Blots were washed and signals were measured by chemiluminescence reagents (Bio-Rad) with imaging system (Bio-Rad). The following antibodies were used: HOXA9 (ab140631, Abcam, Cambridge, MA, USA); BCL2 (ab182858, Abcam). As necessary, blots were stripped and reprobed with β-actin antibody (ab6276, Abcam) as an endogenous control.

Colony formation assay

BM GFP$^+$ cells were sorted and isolated from MLL-AF9-transformed murine AML; BM LSK (Lin$^-$ c-Kit$^+$Sca-1$^+$) cells were isolated from 182WT and 182KO mice (about 8-week-old). Murine GFP$^+$

leukemic blasts and normal LSK cells were plated on murine methylcellulose medium (MethoCult™ GF M3434, Stemcell Technologies, Vancouver, BC, Canada), and colonies were counted 10 days after plating. Human pre-leukemic LSCs ($CD34^+$) were sorted from BM of untreated AML patients and $CD34^+$ HSPCs were sorted from NCs. MOLM-13 and THP1 cells were transduced with overexpression of miR-182 (182OE) or negative control (182NC). These cells were seeded into human methylcellulose medium (MethoCult^{TM} H4434 Classic, Stemcell Technologies) and colonies (>40 cells) were counted 10 days after plating according to manufacturer's protocol.

Cell proliferation and apoptosis assays

Cell proliferation was assessed by CCK8 assay (Beyotime Biotechnology, Nanjing, Jiangsu, China). Apoptosis was measured by Annexin V/7-AAD staining (Invitrogen). Briefly, collected leukemic cells were washed by 1×binding buffer and incubated with 100 μL 1×binding buffer with 5.0 μL Annexin V-APC and 5.0 μL 7-AAD at room temperature for 20 min. Leukemic cells were resuspended with 400 μL 1×binding buffer and analyzed by flow cytometry (CytoFLEX LX).

Luciferase reporter

On the day before transfection, 293T cells were plated on 24-well plates in a concentration of 1×10^5/mL per well. Each well was transiently co-transfected with 100 ng psiCHECK-2 plasmid bearing the wide-type 3'-untranslated region (UTR) of *HOXA9* and *BCL2* or 3'UTR with the mutated miR-182-binding site of *HOXA9* and *BCL2*, together with 60 pmol scramble (SCR), or miR-182 mimics (GenePharma, Shanghai, China). Cell lysates were harvested after transfection for 48 h, and Firefly and Renilla luciferase activities were measured by the Dual-Luciferase Reporter Assay System (Promega, Madison, WI, USA). Firefly luciferase activity was normalized to that of Renilla luciferase for relative luciferase activity for each assay.

Competitive reconstitution assays

Donor cells (Ly45.2, 5×10^5) from 182WT or 182KO mice and equal WT competitors (Ly45.1, 5×10^5) were mixed and xenografted into lethally irradiated recipients (Ly45.1). PB was collected monthly after transplantation for four times. The ratio of CD45.2/CD45.1 was analyzed by flow cytometry (CytoFLEX LX, Beckman-Coulter, Brea, CA, USA). C57BL/6 mice congenic for the CD45 locus (CD45.1 mice were kindly given by Prof. Duan from Shanghai Jiaotong University School of Medicine, Shanghai, China) were used as recipients in the competitive reconstitution assay.

RNA sequencing (RNA-seq)

GFP^+ cells from 182WT and 182KO AML mice were sorted by flow cytometry. Total RNA was extracted by Trizol reagent (Invitrogen, USA) according to the manufacturer's protocol. For library construction, mRNA enrichment, fragmentation, and cDNA synthesis were performed by KAPA Stranded RNA-Seq Library Preparation Kit (Illumina Technologies, San Diego, CA, USA). Sequencing was performed on Illumina HiSeq 4000 sequencing platform after double-stranded cDNA samples were verified with an Agilent 2100 Bioanalyzer (Agilent Technologies, Santa Clara, CA, USA). We used Illumina/Solexa Pipeline Image analysis to analyze base calling and error estimation. To reconstruct the transcriptome, the trimmed reads were mapped to the corresponding reference genome by HISAT2 and StringTie. To analyze GSEA, the negative logarithm of the adjusted *p*-value was first multiplied by the sign of the fold change for each gene to generate pre-ranked gene lists. The enrichment score for each gene set was calculated by input.

Cell cycle

Leukemic cells (2×10^6) were incubated in PBS buffer with propidium iodide (0.04 mg/mL, Invitrogen) and RNase (100 mg/mL) at room temperature for 30 min. Cells were collected by flow

cytometry and FlowJo software v10.0 (Ashland) was performed to produce histograms to assess number of cells in each phase of the cell cycle.

Construction of plasmids

Human and murine pre-miR-182 and its flanking regions were cloned into retroviral vector pMSCV-puro (Clontech, Palo Alto, CA, USA) to construct the plasmids expressing human and murine miR-182. 3'-UTR of *HOXA9* and *BCL2* including miR-182-binding sites were amplified by PCR and cloned into vector psiCHECK-2 (Promega). The mutations on miR-182-binding sites in 3'-UTR of *HOXA9* and *BCL2* were generated by the site-directed mutagenesis kit (Stratagene, Santa Clara, CA, USA). To construct the plasmids expressing HOXA9 and BCL2, we amplified coding sequence (CDS) of *HOXA9* and *BCL2* and inserted them into vector pLVX-puro. All of the primer sequences are shown in Table S2. DNA sequencing was performed to confirm the sequence.

Lentiviral and retroviral production and transduction

For the production of lentivirus and retrovirus, HEK293T cells (4×10^6) were plated in a 10 cm dish one day before transfection. Lentiviral vector LVX- puro (7.5 μg) together with packaging plasmids (1.875 μg MD2G and 5.625 μg PSPA2) and retroviral vector MSCV-puro (5.625 μg) together with packaging plasmids (5.625 μg Gap pol and 3.75 μg VSVG) were co-transfected into 293T cells using 45 μg linear polyethylenimine (Sigma-Aldrich), respectively. The virus was collected from the supernatant at 48 and 72 h after transfection, followed by a 0.45 μm polysulfone filter (Millipore, Billerica, MA, USA). Virus with polybrene (Sigma-Aldrich) were added to leukemic cells and centrifugated at 2000×rpm for 2 h to improve transduction efficiency. Puromycin (1 μg/mL, MCE, Princeton, NJ, USA) was added to the supernatant to select positive clones for at least one week.

DNA methylation detection

Genomic DNA was extracted from leukemia cell lines and BM mononuclear cells (MNCs) from AML patients and NCs by using the DNA Mini kit (QIAGEN, Hilden, Germany) according to the manufacturer's instructions. CpG islands at pri-miR-182 promoter were selected according to the following criteria:1.>200 bp length; 2.cytosine and guanine content >50%; 3.⩾0.60 ratios of observed/expected dinucleotides CpG. Finally, 82 CpG methylation sites on three islands were sequenced. Bisulfite conversion of all unmethylated cytosine to uracil was performed by the EZ DNA Methylation™-GOLD Kit (ZYMO RESEARCH, Irvine, CA, USA), and the samples with bisulfite conversion rate < 98% were first filtered out. We used MethylTarget™ assays as targeted bisulfite sequencing by Genesky BioTech (Shanghai, China) [1]. After target CpG regions were amplified, separated, and purified by Gel Extraction kit (Vazymebiotech, Nanjing, Jiangsu, China), CpG islands methylation assay was performed by Illumina Hiseq/Miseq 2000 according to the manufacturer's protocol.

Flow cytometry analysis

To measure GFP^{+} cells in the murine AML model, peripheral blood (PB) cells were collected and lysed with lysis buffer (BD PharMingen, San Diego, CA, USA). BM cells were isolated by crushing bones from mice and stained with various primary antibodies in staining buffer for 30 min. Antibodies used for LSC/LIC and normal LT-HSC include Streptavidin-APC-R700; Sca-1-PE-Cy7; CD34-APC; c-Kit-PE; CD16/CD32-PerCP-Cy5.5; Mac-1-PE-Cy7; CD135-BV421; (all from BD PharMingen). GMP-like leukemic cells (L-GMP, GFP^{+}Lin^{-}c-Kit^{+}Sca-1^{-}CD34^{+}CD16/32^{+}) were used as the marker for LSC [2]. c-Kit^{+}Mac-1^{+} cells were used as the marker for LIC [3]. Lin^{-}c-Kit^{+}Sca-1^{+}CD34^{-}CD135^{-} cells were used as the marker for long-term hemopoietic stem cells (LT-HSC) in 182WT and 182KO mice [3]. Thymidine analog 5-ethynyl-2'-deoxyuridine (EdU) staining

was performed following protocol (Beyotime Biotechnology). Analysis was performed by flow cytometry (CytoFLEX LX, Beckman-Coulter), and cell sorting was performed by FACS Aria II (Becton Dickinson). Data were analyzed with FlowJo software v10.0 (Ashland, OR, USA).

Hematological and histological analysis

Peripheral blood (PB) counts were measured by a hemacytometer (MINDRAY Medical International Co., Ltd, Shenzhen, China). Murine BM cytospins and PB smears were stained by Wright-Giemsa stain following standard protocols for morphological analysis [4]. Paraformaldehyde-fixed paraffin-embedded sections of murine spleen and liver tissues were subjected to H&E staining by standard protocols.

Producing miR-182 knockout (182KO) mice

The precursor sequence of miR-182 was replaced by the neomycin resistance gene under the control of the phosphoglycerate kinase 1 promoter in a targeting construct. The targeting vector was linearized and transfected into embryonic stem (ES) cells. Recombinant ES clones were selected and injected into blastocysts to generate male chimeras. Heterozygous and homozygous mice were obtained after five generations of backcrossing and were confirmed using genotyping and northern blotting [5]. Genotype of 182KO mice was performed according to previous report [5]. Briefly, DNA was extracted from the tail of 182WT and KO mice and PCR was performed by standard procedure. Primers of PCR for genotype were indicated in Table S2.

Supplemental references

1. Pu W, Wang C, Chen S, Zhao D, Zhou Y, Ma Y. et al. Targeted bisulfite sequencing identified a panel of DNA methylation-based biomarkers for esophageal squamous cell carcinoma (ESCC). Clin Epigenetics. 2017; 9: 129.
2. Iwasaki M, Liedtke M, Gentles AJ.Cleary ML. CD93 Marks a Non-Quiescent Human Leukemia Stem Cell Population and Is Required for Development of MLL-Rearranged Acute Myeloid Leukemia. Cell Stem Cell. 2015; 17: 412-21.
3. Zhang Y, Xia F, Liu X, Yu Z, Xie L, Liu L. et al. JAM3 maintains leukemia-initiating cell self-renewal through LRP5/AKT/beta-catenin/CCND1 signaling. J Clin Invest. 2018; 128: 1737-51.
4. Song MG, Gao SM, Du KM, Xu M, Yu Y, Zhou YH. et al. Nanomolar concentration of NSC606985, a camptothecin analog, induces leukemic-cell apoptosis through protein kinase Cdelta-dependent mechanisms. Blood. 2005; 105: 3714-21.
5. Jin ZB, Hirokawa G, Gui L, Takahashi R, Osakada F, Hiura Y. et al. Targeted deletion of miR-182, an abundant retinal microRNA. Mol Vis. 2009; 15: 523-33.

Table S1. the clinical characteristics of 96 AML patients

Characteristic	All patients N (%)	*P* value
Overall		
Gender		0.36
Male	52(54.1)	
Female	44(45.9)	
Age (range)	53(19-72)	
FAB subtype		
M1	8(8.3)	
M2	16(16.7)	
M3	8(8.3)	
M4	30(31.2)	
M5	32(33.3)	
Unclassified	2(2.0)	
Cytogenetics		
Normal karyotype	35(36.5)	
t(15;17)	8(8.3)	
t(8;21)	13(13.5)	
t(11q23)	9(9.4)	
Inv(16)	10(10.4)	
+8	8(8.4)	
+11	3(3.1)	
+21	2(2.1)	
Complex karyotype	6(6.3)	
Not available	2(2.1)	
Molecular genetic abnormality		
PML-RARα	8(8.3)	
AML1-ETO	13 (13.5)	
CBF-MYH11	10(10.4)	
MLL-arrangements	9(9.4)	
WT1 mutation	11(11.4)	0.37
FLT3 mutation	12(12.5)	0.27
CEBPα mutation	18(18.8)	0.35
TET2 mutation	9(9.4)	0.18
MLL-PTD mutation	3(3.1)	0.053
c-Kit mutation	8(8.3)	0.09
NPM1 mutation	5(5.2)	0.27

Table S2. The sequences of primers for qRT-PCR and construction of plasmids

Genes	Sequences
miR-182-5P-RT	GTC GTA TCC AGT GCA GGG TCC GAG GTA TTC GCA CTG GAT ACG ACA GTG TGA G
miR-182-5P-L	GAT TTG GCA ATG GTA GAA CTC AC
miR-182-5P-R	GTG CAG GGT CCG AGG T
miR-183-5P-RT	5′-GTC GTA TCC AGT GCA GGG TCC GAG GTA TTC GCA CTG GAT ACG ACA GTG AA-3′
miR-183-5P-L	5′-CGC GTA TGG CAC TGG TAG AA-3′
miR-183-5P-R	5′-AGT GCA GGG TCC GAG GTA TT-3′
miR-96-5P-RT	5′-GTC GTA TCC AGT GCA GGG TCC GAG GTA TTC-3′ GCA CTG GAT ACG ACA GCA AA-3′
miR-96-5P-L	5′-GCG TTT GGC ACT AGC ACA TT-3′
miR-96-5P-R	5′-AGT GCA GGG TCC GAG GTA TT-3′
HOXA9-L	5′-TAC GTG GAC TCG TTC CTG CT-3′
HOXA9-R	5′-CGT CGC CTT GGA CTG GAA G-3′
BCL2-L	5′-GGT GGG GTC ATG TGT GTG G-3′
BCL2-R	5′-CGG TTC AGG TAC TCAG TCA TCC-3′
GAPDH-L	5′-CCG GGA AAC TGT GGC GTG ATG G -3′
GAPDH-R	5′-AGG TGG AGG AGT GGG TGT CGC TGT T -3′
β-actin-L (mouse)	5′-GGC TGT ATT CCC CTC CAT CG-3′
β-actin-R (mouse)	5′-CCA GTT GGT AAC AAT GCC ATG T-3′
Hoxa6-L (mouse)	5′-CGG CCA GGA CTC CTT CTT G-3′
Hoxa6-R (mouse)	5′-CCG AGT TGG ACT GTT GGT AAA A-3′
Hoxa9-L (mouse)	5′-CCC CGA CTT CAG TCC TTG C-3′
Hoxa9-R (mouse)	5′-GAT GCA CGT AGG GGT GGT G-3′
Hoxa10-L (mouse)	5′-CCT GCC GCG AAC TCC TTT T-3′
Hoxa10-R (mouse)	5′-GGC GCT TCA TTA CGC TTG C-3′
Jmjd1c-L (mouse)	5′-CAC CCG CAC CAT GAT CGT TAT-3′
Jmjd1c-R (mouse)	5′-CTT CGC CGT GAT GTA ATG CC-3′
Meis1-L (mouse)	5′-GCA AAG TAT GCC AGG GGA GTA-3′
Meis1-R (mouse)	5′-TCC TGT GTT AAG AAC CGA GGG-3′
Cd34-L (mouse)	5′-AAG GC TGG GTG AAG ACC CTT A-3′
Cd34-R (mouse)	5′-TGA ATG GCC GTT TCT GGA AGT-3′
Mac-1-L (mouse)	5′-GGC TCC GGT AGC ATC AAC AA-3′
Mac-1-R (mouse)	5′-ATC TTG GGC TAG GGT TTC TCT-3′
*pMSCV-miR-182-L	5′-GAA GAT CTT AGG GAT GGT GTC TGC TCC A-3′
*pMSCV-miR-182-R	5′-GGA ATT CAG AGT GTC ACT TCC AGC TGC-3′
*pLVX-miR-182-L (mouse)	5′-CGG AAT TCA GAG TGC ACC CAT TCC CAA G-3′
*pLVX-miR-182-R (mouse)	5′-CGG GAT CCT CAC CGA GAA GAG GTC GAC T-3′
*pLVX-HOXA9-L	5′-CCG CTC GAG GCC ACC ATG GCC ACC ACT GGG GCC CT-3′

*pLVX-HOXA9-R	5′-ATA AGA ATG CGG CCG CTC ACT CGT CTT TTG C TC GG T-3′
*pLVX-BCL2-L	5′-CCG CTC GAG GCC ACC ATG GCG CAC GCT GGG AGA AC-3′
*pLVX-BCL2-R	5′-ATA AGA ATG CGG CCG CTC ACT TGT GGC CCA GAT AGG-3′
*SiCHECK-2-HOXA9-L	5′-CCG CTC GAG ACC GAG CAA AAG ACG AGT GA-3′
*SiCHECK-2-HOXA9-R	5′-ATA AGA ATG CGG CCG CTA GAA AAG AAT CAA TAT ATT-3′
*SiCHECK-2-BCL2-L	5′-CCG CTC GAG ACC ATG AAA CAA AGC TGC AGG -3′
*SiCHECK-2-BCL2-R	5′-ATA AGA ATG CGG CCG CAC AGG AGT TTT GAT GGG ACT GT-3′
Primer1 of miR-182 genotype	5′-GGA CCA TAC AGG CCG AAG GAC-3′
Primer2 of miR-182 genotype	5′-CCT TCT ATC GCC TTC TTG ACG AGT TC-3′
Primer3 of miR-182 genotype	5′-CCC AAG TCC TTT TCA CCG AGA AGA G-3′

* Primers for constructing plasmids.

Table S3. Limiting dilution assay of MLL-AF9-transduced 182WT and 182KO AML mice

Dose	182WT (response/total)	182KO (response/total)	*P* value
30	3/6	5/6	
90	4/6	6/6	
180	6/6	6/6	
LSC frequency	1 in 56	1 in 16	0.02

The numbers of response mice mean that the recipient mice develop full-blown leukemia and die within six months after transplantation.

Table S4. miR-182 expressions in AML patients with CpG island 3 methylation or unmethylation

AML patients	CpG island 3 methylation %	miR-182 expression
*5	8.1	9.59
*36	14.8	5.62
*38	13.4	1.66
*44	10.7	0.17
*55	6.0	0.05
*62	17.6	0.80
*73	18.3	8.29
*105	16.7	4.67
*109	15.9	1.49
*110	7.0	7.11
*113	12.2	8.30
#1	55.0	0.79
#3	62.7	1.87
#4	46.7	6.81
#20	33.7	0.27
#22	24.1	0.09
#26	30.2	0.14
#43	25.9	0.20
#56	30.1	0.15
#58	27.4	1.46
#64	46.0	0.09
#66	37.6	0.80
#67	28.5	0.81
#70	21.2	0.28
#72	20.9	1.75
#74	21.1	0.15
#77	36.9	1.15
#80	70.1	0.07
#82	65.7	0.34
#101	25.9	1.66
#115	56.9	0.06

*CpG island 3 methylation% < 20%; #CpG island 3 methylation% > 20%

Figure S1

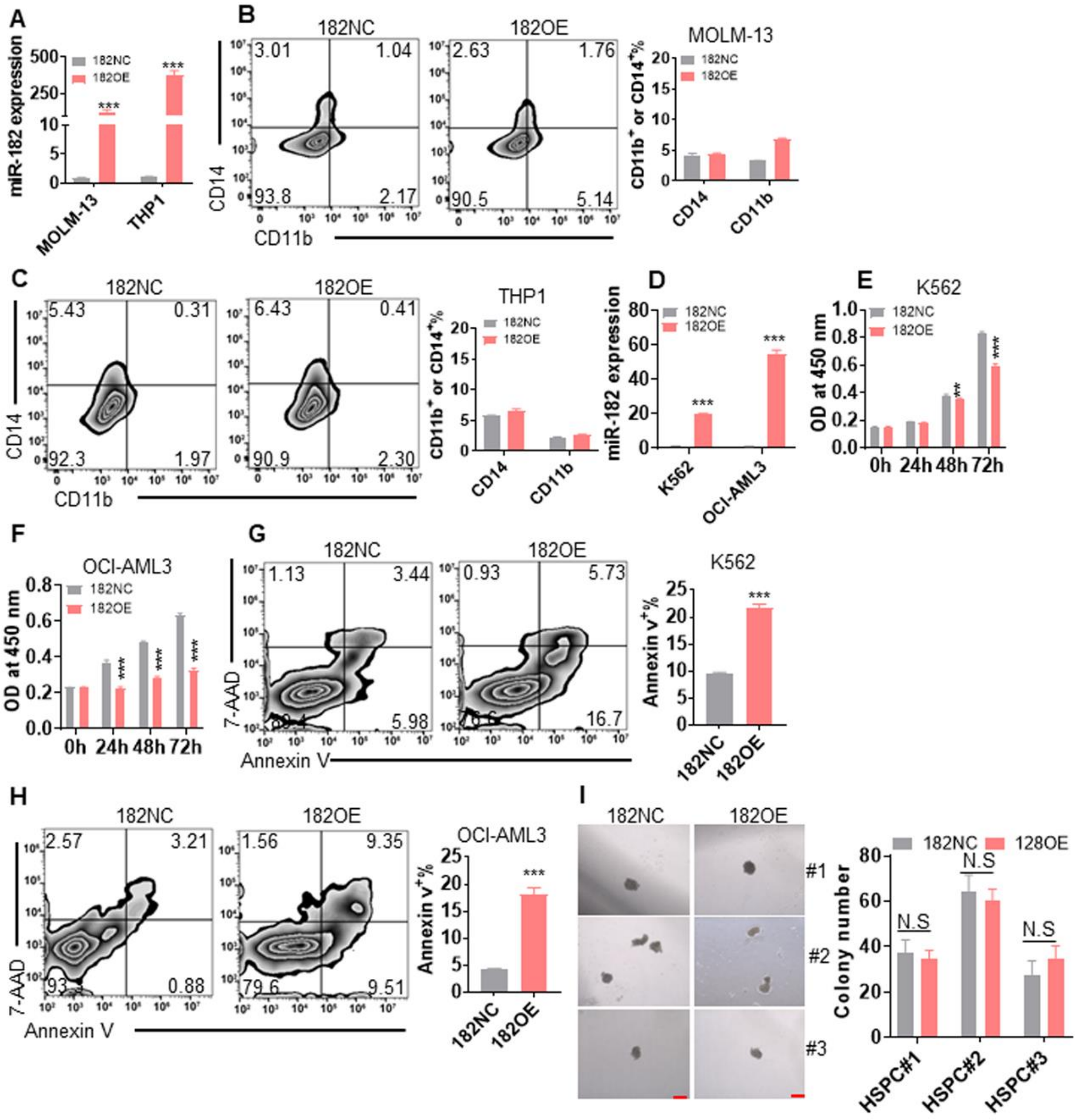

Figure S1. Effects of overexpression of miR-182 on AML blasts and normal HSPCs. (**A**) The expression of miR-182 was measured in MOLM-13 and THP1 cells transduced with MSCV-puro-NC (182NC) or MSCV-puro-miR-182 (182OE). (**B and C**) CD14 and CD11b were measured in MOLM-13 (**B**) and THP1 cells (**C**) transduced with 182NC or 182OE. Shown are the representative plots (left) and statistical analysis of $CD14^+$ and $CD11b^+$ cells (right). (**D**) The expression of miR-182 was measured in K562 and OCI-AML3 cells transduced with 182NC or 182OE. (**E and F**) CCK8 was measured in K562 (E) and OCI-AML3 cells (F), which were transduced with 182NC or 182OE. (G and H) Apoptosis was measured by staining Annexin V and 7-AAD in K562 (G) and

OCI-AML3 cells (**H**), which were transduced with 182NC or 182OE. (**I**) Colony number was counted in three normal $CD34^+$ HSPCs transduced with 182OE or 182NC. HSPCs (1×10^3/dish) were plated in methylcellulose medium for ten days to count colony number. Shown are the representative pictures (left) and statistical analysis of the colony (right). Bar scales represent 200 μm. **$P < 0.01$; ***$P < 0.001$.

Figure S2

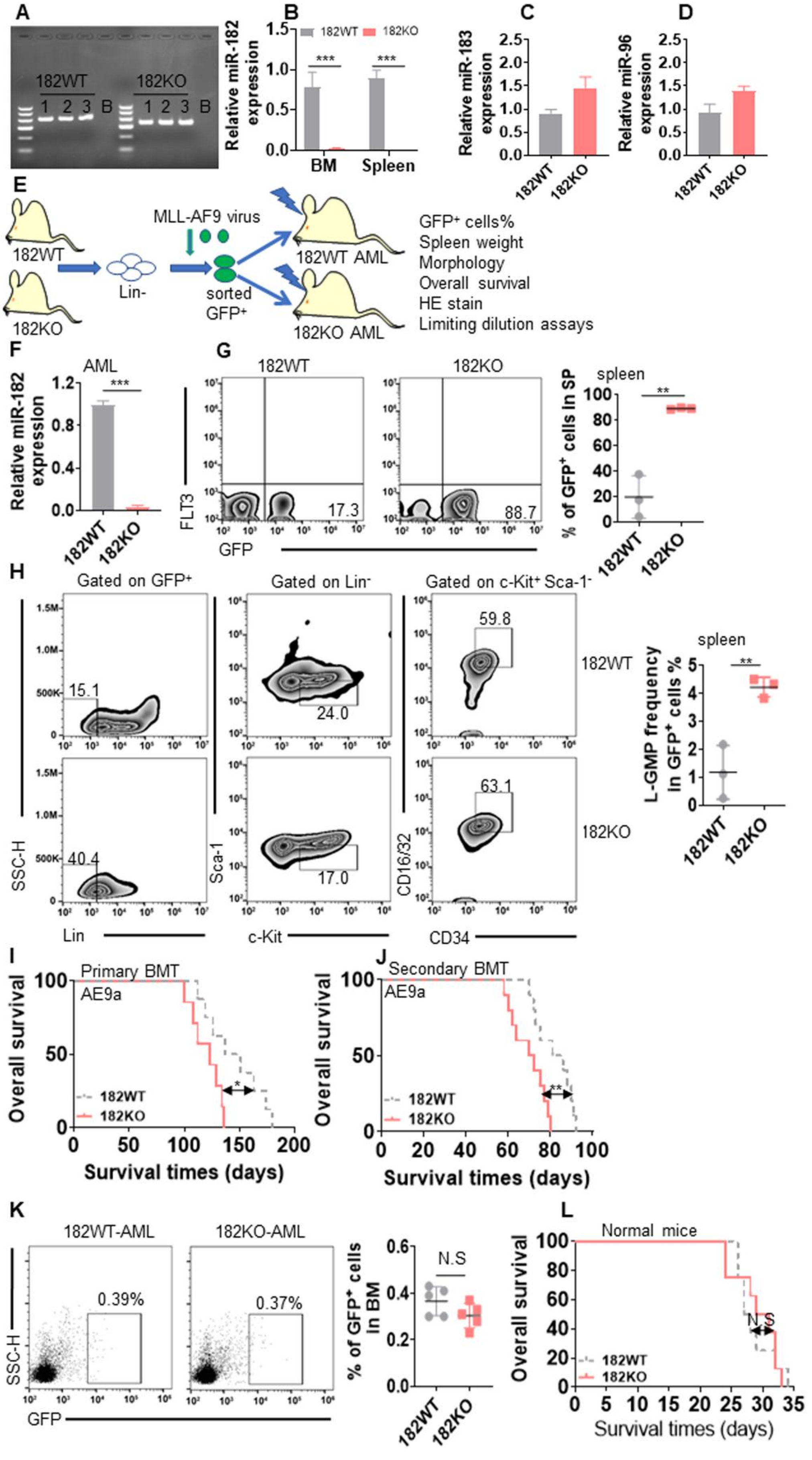

A
182WT
182KO
1 2 3 B
1 2 3 B
B
182WT
182KO
Relative miR-182 expression
BM
Spleen
C
Relative miR-183 expression
182WT
182KO
D
Relative miR-96 expression
182WT
182KO
E
MLL-AF9 virus
182WT
182KO
Lin-
sorted GFP+
182WT AML
182KO AML
GFP+ cells%
Spleen weight
Morphology
Overall survival
HE stain
Limiting dilution assays
F
AML
Relative miR-182 expression
182WT
182KO
G
182WT
182KO
FLT3
GFP
17.3
88.7
spleen
% of GFP+ cells in SP
182WT
182KO
H
Gated on GFP+
Gated on Lin-
Gated on c-Kit+ Sca-1-
15.1
24.0
59.8
182WT
40.4
17.0
63.1
182KO
SSC-H
Sca-1
CD16/32
Lin
c-Kit
CD34
spleen
L-GMP frequency in GFP+ cells %
182WT
182KO
I
Primary BMT
AE9a
Overall survival
182WT
182KO
Survival times (days)
J
Secondary BMT
AE9a
Overall survival
182WT
182KO
Survival times (days)
K
182WT-AML
182KO-AML
0.39%
0.37%
SSC-H
GFP
% of GFP+ cells in BM
N.S
182WT
182KO
L
Normal mice
Overall survival
N.S
182WT
182KO
Survival times (days)

Figure S2. Depletion of miR-182 develops the progression in MLL-AF9-induced AML mouse model. (**A**) Genotyping PCR for miR-182-knockout mice (182KO) or WT mice (182WT). (**B**) miR-182 expression was measured by qRT-PCR in BM and spleen cells from 182WT mice and 182KO mice. (**C and D**) miR-183 and miR-96 expressions were measured by qRT-PCR in BM cells from 182WT mice and 182KO mice. (**E**) Schedule for MLL-AF9-induced AML mouse model. (**F**) miR-182 expression was measured in GFP^+ cells from 182WT and 182KO AML blasts. (**G**) The percentages of GFP^+ cells were measured in spleen from MLL-AF9-transformed murine AML with 182WT (n = 3) and 182KO (n = 3). Shown are the representative plots (left) and statistical analysis of GFP^+ cells (right). (**H**) The frequencies of L-GMP cells ($Lin^-c\text{-}Kit^+Sca\text{-}1^-CD34^+CD16/32^+$) were measured in spleen GFP^+ cells from murine AML with 182WT (n = 3) and 182KO (n = 3). Shown are the representative flow cytometry plots (left) and statistical analysis of L-GMP cells (right). (**I and J**) Overall survival time for recipient mice receiving 182WT- and 182KO-AML1-$ETO9a^+$ (AE9a) BM cells upon the primary (n = 8 for WT and n = 7 for KO mice) and secondary transplantation (n = 10 for WT and KO mice). (**K**) GFP^+ blasts isolated from 182WT and 182KO murine AML blasts were injected into the lethally irradiated wild-type mice (2×10^6/mice, n = 5). The homed cells (GFP^+) were measured in BM at 16 h after transplantation. Shown are the representative flow cytometry plots (left) and statistical analysis of GFP^+ cells (right). (**L**) GFP^+ blasts from MLL-AF9-transformed AML mice were transplanted into normal 182WT (n = 8) and 182KO mice (n = 8), followed by counting OS time. *$P < 0.05$; **$P < 0.01$; ***$P < 0.001$. Using log-rank test for survival time. N.S: not significant.

Figure S3

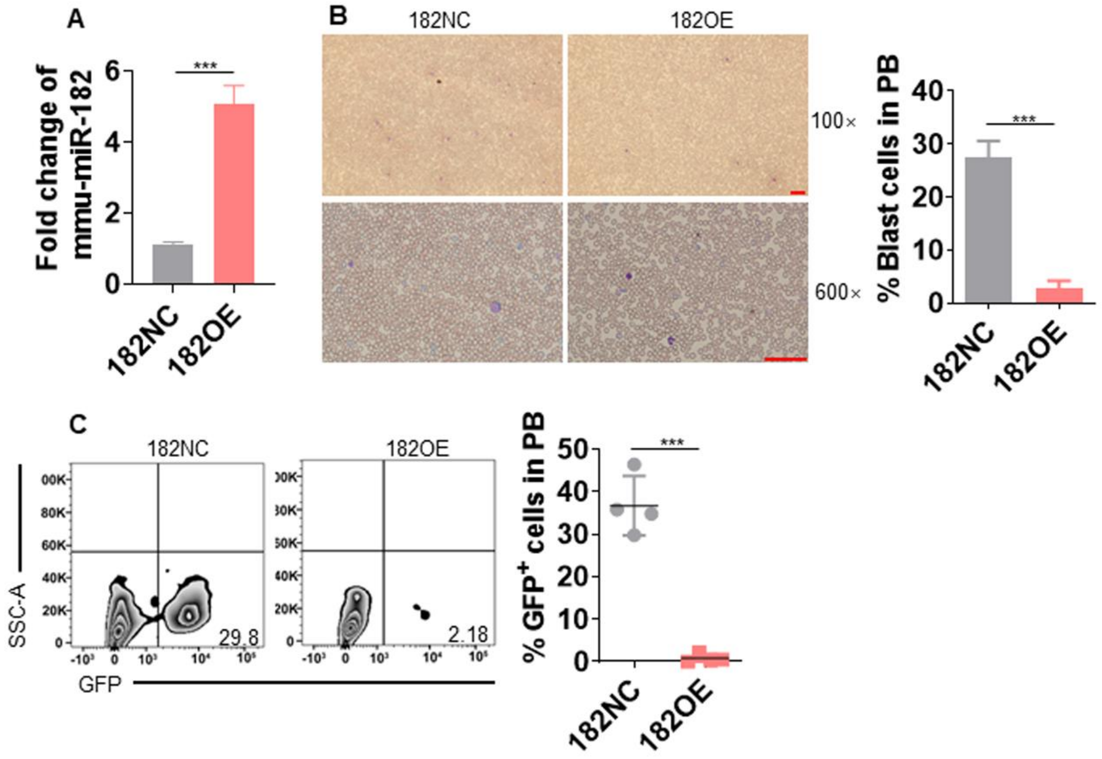

Figure S3. Overexpression of murine miR-182 (182OE) delays the leukemogenesis in MLL-AF9-transformed AML mice. (**A**) Relative expression of miR-182 in BM GFP$^+$ cells from recipients receiving 182NC AML cells compared with those receiving 182OE AML cells. (**B**) The representative images of PB smears by Wright-Giemsa stain in recipients receiving 182NC AML cells compared with those receiving 182OE AML cells (left) and statistical analysis of leukemic blasts (right). Bar scales represent 50 μm. (**C**) GFP$^+$ cells were measured in PB from recipients receiving 182NC AML cells compared with those receiving 182OE AML cells. Shown are the representative plots (left) and statistical analysis of GFP$^+$ cells (right). n = 4 for 182NC and 182OE. ***$P < 0.001$.

Figure S4

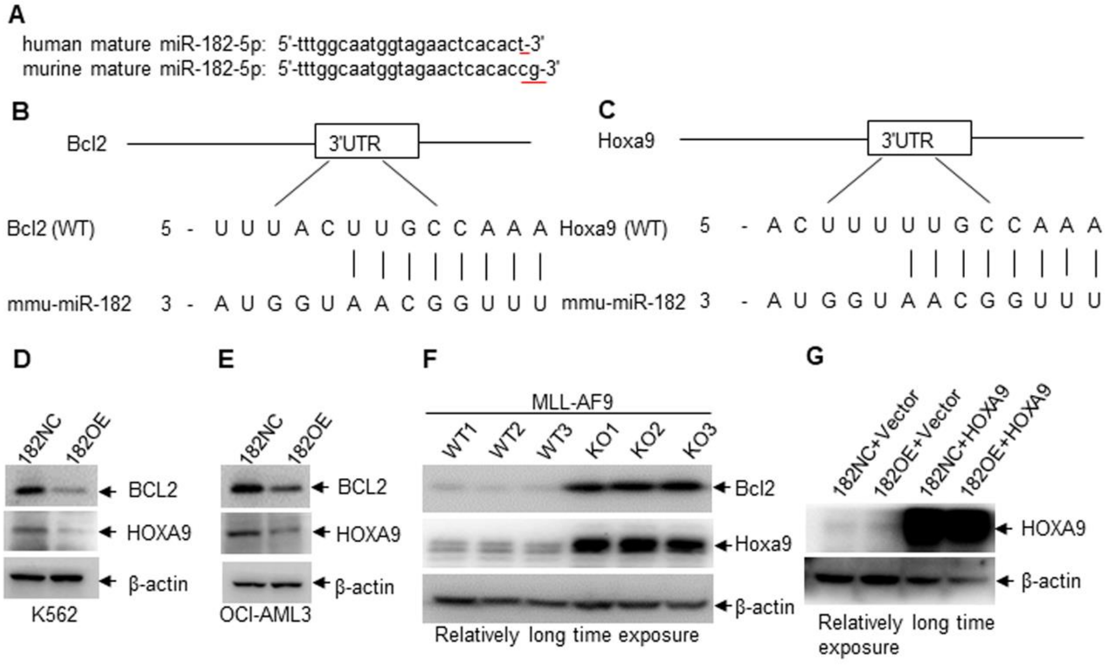

Figure S4. miR-182 directly targets *BCL2* and *HOXA9*. (**A**) Comparison with human miR-182 and murine miR-182 sequences. (**B and C**) Schematic of putative binding sites for murine miR-182 in 3'-UTR of *Bcl2* and *Hoxa9*. (**D and E**) The protein expressions of BCL2 and HOXA9 were measured in K562 and OCI-AML3 cells transduced with 182OE or 182NC. (**F**) The protein levels of Bcl2 and Hoxa9 were measured by Western blot with relatively long time exposure in MLL-AF9-transformed murine AML blasts with 182WT or 182KO. (**G**) The protein expressions of HOXA9 were determined by Western blot with relatively long time exposure in 182OE-and 182NC-transduced THP1 cells, following by transduction with LVX-HOXA9 or LVX-NC (Vector), respectively.

Figure S5

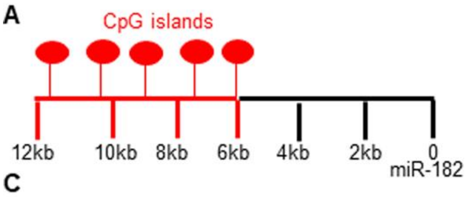

B

CpG	CpG Island Start	CpG Island End	CpG Island Length
1	281	736	456
2	1840	3381	1542
3	3387	4081	695

C

tccagCGagagggaatggggcaattgccagaCGgacattggatctgcagaaggaggggtaggatacaCGCGggcttaggatg
CGctggacaggccagcaaagtgaggaccccaggtCGtcctgagccacctcttccccCGcctgagccctgagcCGtaagggatcc
agggtatcaggtcccCGgggcCGccccctcacCGtcCGagtccagctcCGgttacctgcctggcctgctccttaaaatatgtatga
ggcccttgCGggtgcagtttCGCGgccctccCGgagtggggctggggactcagggtgCGaggggagctgagggggaagga
agCGaCGCGagctcccCGgcCGgggccttctCGactttcagcagCGcatttaagaaaaggtggaaatCGgatctgtctCGg
CGgCGccccagcccCGccCGctcCGaCGgcCGgacctCGCGgcccagCGgctagctttctgctgCGCGgtgtctggttc
CpG1
tCGacagcaggtgaatgggccagggCGgggCGccCGgagCGCGgggggCGgggCGCGCGttataaaggggagcag
caggcCGCGggggccactCGcagtcCGcaaggcctgCGcttccCGctttgCGgtcctgggcacaccCGgccCGggttgcc
cagcatCGcatgggtctgaggaccaggtagggCGCGggtgcagagacagccagggaggctCGaggCGacctagatgggCG
ggggaatgctagtaggaCGgggagggttaatgtggagctatcaccccttttggccCGttgggctctgtgaCGtgctcattatctcCG
ctgtcCGggttctCGctcacCGtgcccatccctggctgcatggggagaggggcccCGaggccctgagactCGtccagttatccacct
ggggtgggaatcttgcttccCGatccatctggctgCGacatctaCGgcttcagcaggccctgccCGggagtCGggcatattgaagg
gttCGtatccttgcCGtcacccttggcccctccCGgctCGgcaggagctgggggtggggaaactatagatttccCGggcccCGcttc
cactgcctcacccccttcttttgctCGagcccaagacaaggtgttaaCGgtcttcaagtgcaagggttaggtttggtgggagcataggcta
agcaccaccctcattttacaggggcttctagggtgccagatctgCGggggtCGattgggaagaactagaagctaaaaaggagaaggg
CGgattaaCGttttattttaatctggttttgagctgcCGttgcCGtctggggCGgcCGggCGgggCGgagctgggactgccaagg
gcagccaaagcagcagggagggagggggCGctcCGgtgctcaggatgctggggaagaaggaagaggccaccccaaCGctga
cagcttggaggacctagcccctgccccCGgtcccttctgaatgctcCGggtggtgtcagaccaaaggaCGgttgagaaggatctaat
cCGCGtcCGCGccccagcccCGgctggcccCGccccctccCGagggagccactgtCGcagtggggggagggggagatgg
gaaCGggtctggggggCGgctcccctccccctccctctcttgggacagacagcctttgttccCGgCGgCGgCGgcagCGgtgg
ccagacacctgtgaggcCGcagaactggcCGagggtgggCGtgtgaggacatggaggccccttgtccccattccagccctgCGcc
cctgggtcCGggtgCGccccctgagacaggtcagccttgagcacctttctgCGgggagactgcagagatCGtccagtggcctaC
GgtgCGccccagctcccaaCGcacttcccctccccctcccagggtCGgatttccaagcaCGccaCGccacacacacctCGgc
tagctcCGggctcagagggCGctggggtggcagggCGtcCGggCGcacctCGgctagCGgcaccctagccCGagggcag
gaggCGgggatagaaggCGggCGaCGggaccccttggggccagaccCGgcCGggttgggcaagcaggCGgggctccC
GgCGtctcCGcagtccCGgaCGggCGtgccctgCGcCGCGcaccaacCGgagccacattcaaggtgaatcatcccCGg
ccCGcccccctgcccctcccCGcctCGCGgcacctgagccaatggCGctgCGCGaggggCGggCGgggccCGgggCG
gggggCGgtaaaaccctcctggagtcattctctgtgcactcCGtCGagccccCGCGcagcCGtgcCGgagcCGcagggcct
cCGtccagcCGCGCGgtcCGggcagccCGCGccccctgttcttcCGgtcctCGggcccaggtacccctctggacctccccC
GcctgcagCGCGgaggCGgctcctcttcaggtggcCGggcCGgggccttcccCGgcagacctgtcaccCGacacccagga
agCGCGCGgcCGggtccCGgctcccctttgttccccCGctgggctCGgCGctcCGgCGcCGccccctctCGCGgggc
agCGgaggtttgggccCGggcCGggCGCGcttcaagctcCGtccatgCGgctctttccaaacCGgggcCGtttttgcctggctt
CpG2
ctctggtgtggactcCGCGCGgagctgagaggCGgctgtgCGcccagcctggagcCGgCGcaggtgagtgtctggggcCGg
gagCGggggggCGggcCGggCGgagccagagCGgggctcagtagaggaggCGgaggCGggCGccCGggacCGg
cCGcactagaaccCGggtgcagggcagaagtccCGgCGcagctggctttCGggatgaCGCGgcagtgcctactCGctcttc
CGCGccttcaggCGgaggCGcccaCGgggCGcctgggtccCGggtcCGccagtggCGtctgcccagCGcccagCGct
gttccCGggCGggggCGggggCGggagCGgctgCGgtcCGagtgCGgcCGgtgcttgCGaggtgcctaccCGCGag
gagtttggctggggtCGggctgCGtttCGcCGccctggtgggctcaggCGgaagCGccaggctgggagggggctgCGgagag
CGcCGgggcccCGgagctggcccctccagCGtcttgtcagggctgCGCGgctggttCGaggctgcttcCGtggccCGgagc
CGcccCGcccagtctctgggggaaagCGgctctcagacctccCGtgtgcctccacttCGCGgccactcatccCGggaacccC
GgCGcaCGCGaatgagacacCGttcccCGccctcaactgccccattcctgttaatagCGctagtggcacccaggttcccagccc
ttccccaagatCGgCGgctggaCGgcttgcctctggCGcctcacCGgtcagCGcatcccaggactatgggcagtgagggtgtca
ccCGggtcaggggtCGccCGattccaCGgtgtcctccaggctgagccattCGtttgctcttccaaccCGggctccCGgacacC
GtgtacagtaataccCGcCGgggggcaggggcactgaggcCGgctctgagCGattagggcCGagggCGggCGgtgcCGta
atCGacctccccacccccacaggcaaggtgCGgCGgCGcctcccaggtttgCGccCGtcttCGcaaagctcctgagggCGa
aaatcctgCGagccaggtggCGgattctCGggcCGggtcactcagctctaCGggtgCGcatcaccctctacctgtcacccagtga
gccCGacCGatgggcacCGagtCGgCGgatctctgatcCGaggcCGagccttCGgagaaatctgtcctagtggcctcatCGt
CpG3
ggcctgagCGaaCGcaaaggaacctgccccaagcccagctctcagcctCGacccccacattcccCGCGCGgctctCGgttC
GgacctgggctCGgagCGgcagcctccaccactgctCGtttctttacCGctcaaggcCGccccttcatgctcctgCGggggagtta
gCGggacctctggagtCGgccttaCGcaaagaagagaaattctaggatgcCGgcctcacttgtgaggatttCGtttattctttttctcct
acCGgggcccagtggctcttggagtgagc

Figure S5. Hypermethylation of miR-182 promoter in leukemic blasts. (**A**) The regions indicating the CpG islands at putative miR-182 promoter. (**B**) Table indicating the number of CpG islands and the length of CpG islands. (**C**) The detailed information of three CpG islands.

Figure S6

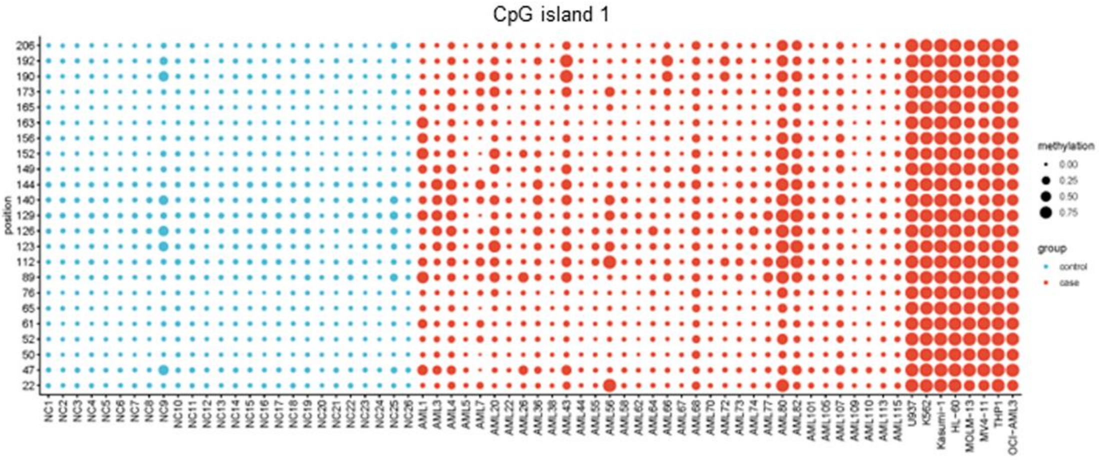

Figure S6. The detailed methylation information of CpG island 1 in normal controls (NCs), primary untreated AML blasts, and AML cell lines.

Figure S7

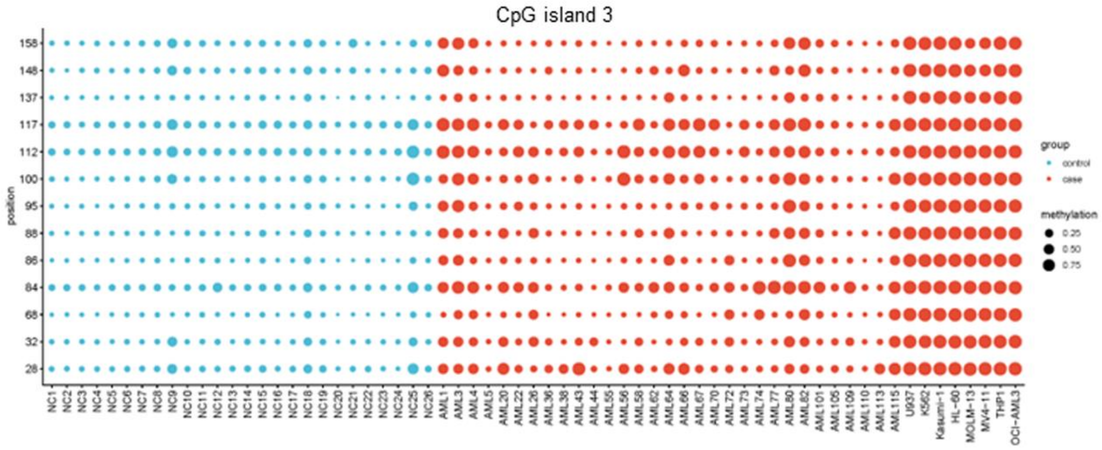

Figure S7. The detailed methylation information of CpG island 3 in NCs, primary AML blasts, and AML cell lines.

Figure S8

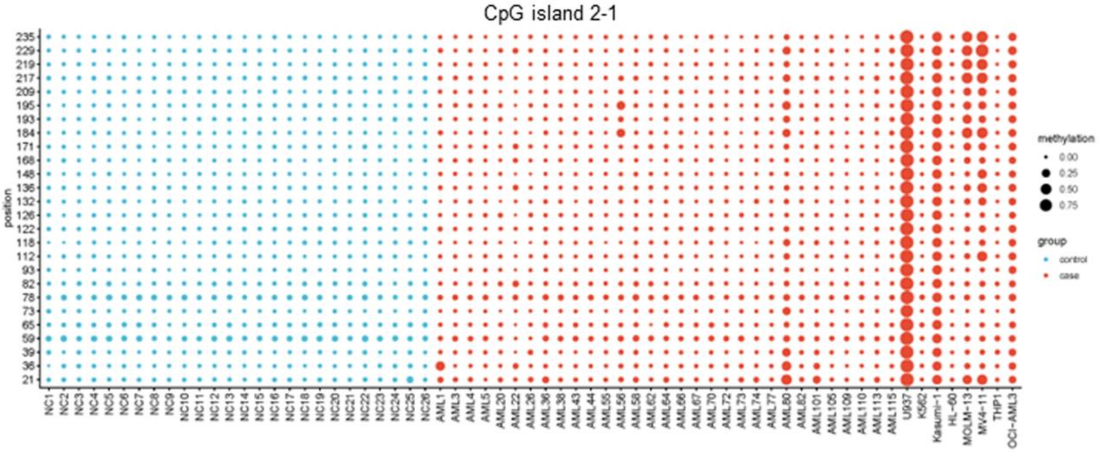

Figure S8. The detailed methylation information of CpG island 2-1 in NCs, primary AML blasts, and AML cell lines.

Figure S9

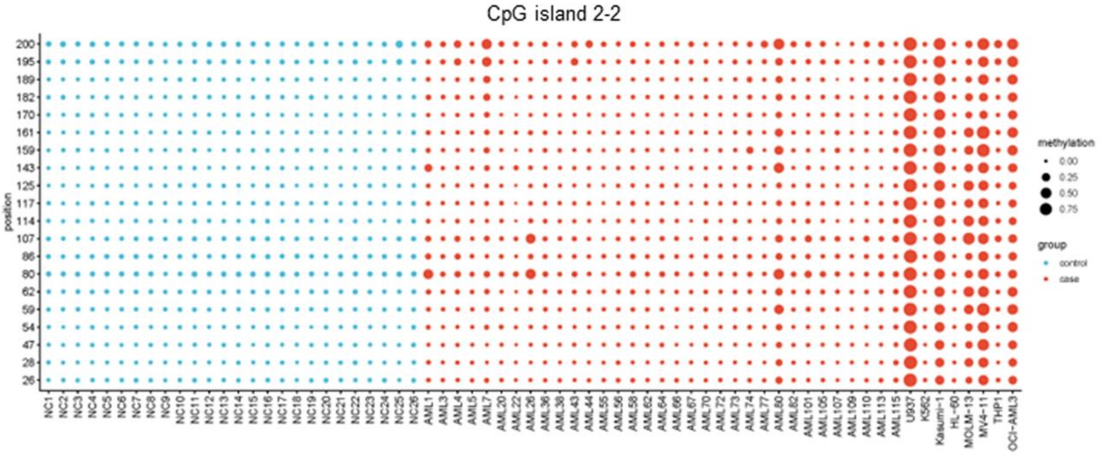

Figure S9. The detailed methylation information of CpG island 2-2 in NCs, primary AML blasts, and AML cell lines.

Figure S10

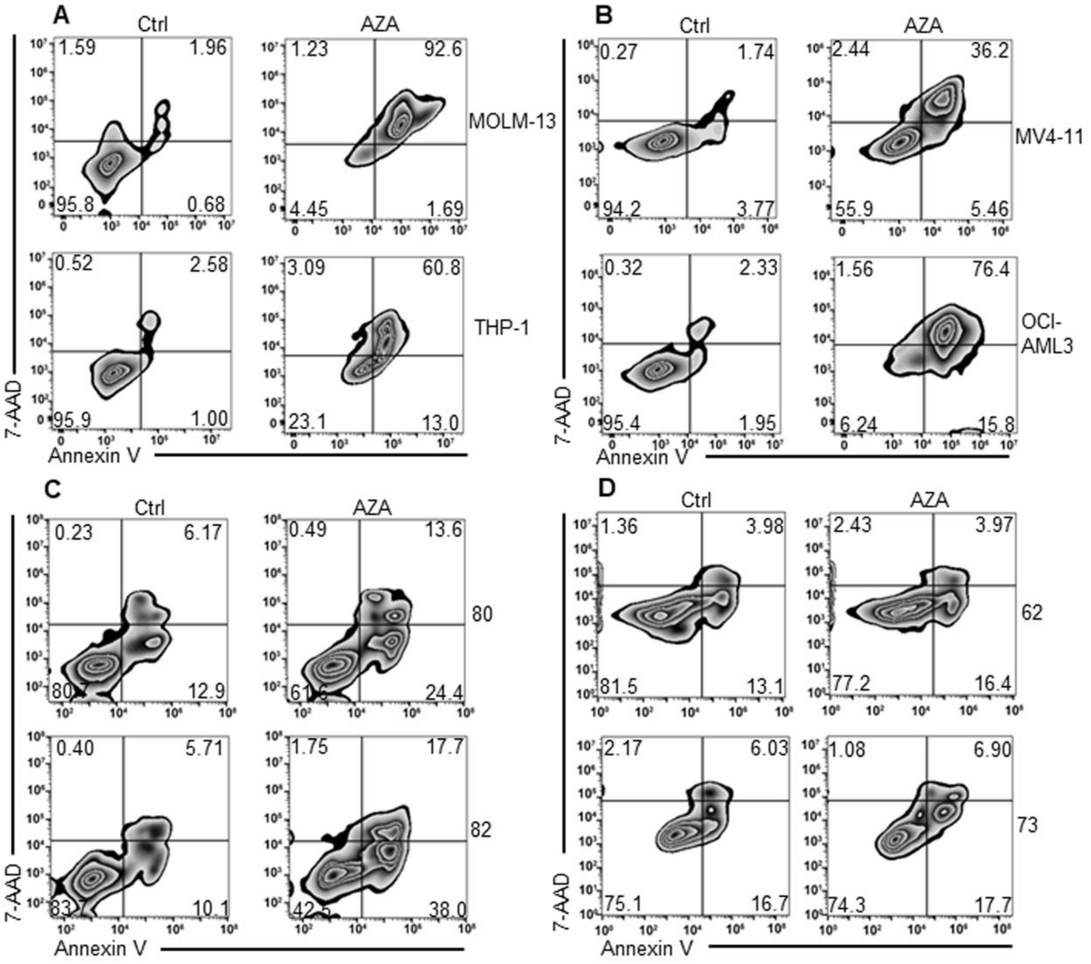

Figure S10. AZA induces apoptosis in leukemic cells. (**A and B**) Apoptosis was measured by Annexin V/7-AAD staining in four leukemic cell lines treated with AZA (5 µM) or not for four days. Representative flow cytometry plots were shown. (**C and D**) Apoptosis was measured in primary AML blasts (80, 82, 62, and 73) treated with AZA (5 µM) or not for four days. Representative flow cytometry plots were shown.
